# Supplementary figures and images for: CCDC66 regulation of cytoskeleton and cilia stability is important for signaling and epithelial organization
Source: PLoS Biol. 2025 Jul 29;23(7):e3003313. doi: 10.1371/journal.pbio.3003313 (PMC12324684; doi:10.1371/journal.pbio.3003313)

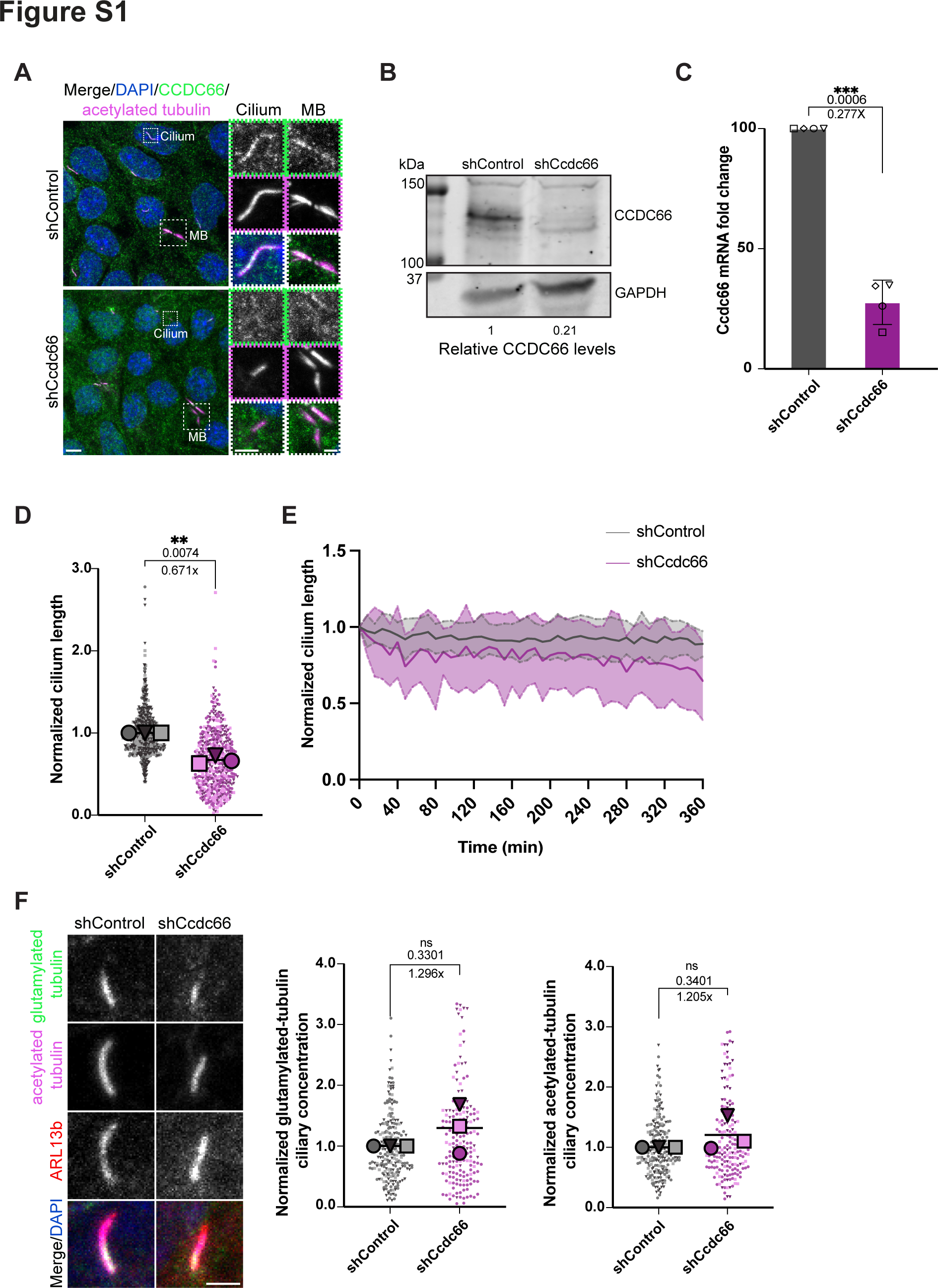

Supplement: S1 Fig — (A–C) The CCDC66 protein was successfully depleted from IMCD3 cells. Control and CCDC66-depleted cells were serum starved for 48 h, fixed with 4% PFA and stained against CCDC66 with homemade antibody, anti-acetylated-tubulin, and DAPI. Scale bars: 5 µm. Insets show 3× magnifications of the cilia and midbody (MB) signals. Scale bar 2 µm (B) western blot analysis of cell lysates from control and CCDC66-depleted samples using homemade CCDC66 antibody and mouse anti-GAPDH as loading control. Represented is fold change of band intensities normalized to loading control of displayed experimental replicates. n = 5 (C) qPCR analysis of mRNA isolated from control and CCDC66-depleted cells with primers recognizing C-terminal internal region and Gapdh primers as normalization control. Box plot shows mean ± SD. n = 4 (One sample t test p value **p = 0.0006) (D–F). (D) Normalized data of cilia length quantification in Fig 1D. Normalization is performed by dividing individual length values with the average control value of experimental replicate (E) Normalized data of cilia length quantification in Fig 1E. Normalization is performed by dividing individual values with the average control value of experimental replicate at t = 0. (F) Quantification of ciliary axoneme PTMs in control and CCDC66-depleted cells, serum starved and stained against acetylated and polyglutamylated-tubulin and ARL13b to mark cilia. Measured are ciliary integrated densities = mean intensity/area, to better represent levels of PTMs in cilia of varying size. Super plot represents normalized individual experimental values and means ± SD of 3 independent experiments. n > 150 cilia for each conditioned and PTM. (p value of Welch’s t tests ns: not significant). Statistical analysis is performed on the means of 3 experimental replicates. The data underlying the graphs shown in the figure can be found in S1 Data. MB, midbody; mRNA, messenger ribonucleic acid; GAPDH, Glyceraldehyde 3-phosphate dehydrogenase; PTM, pos [file pbio.3003313.s001.tif]

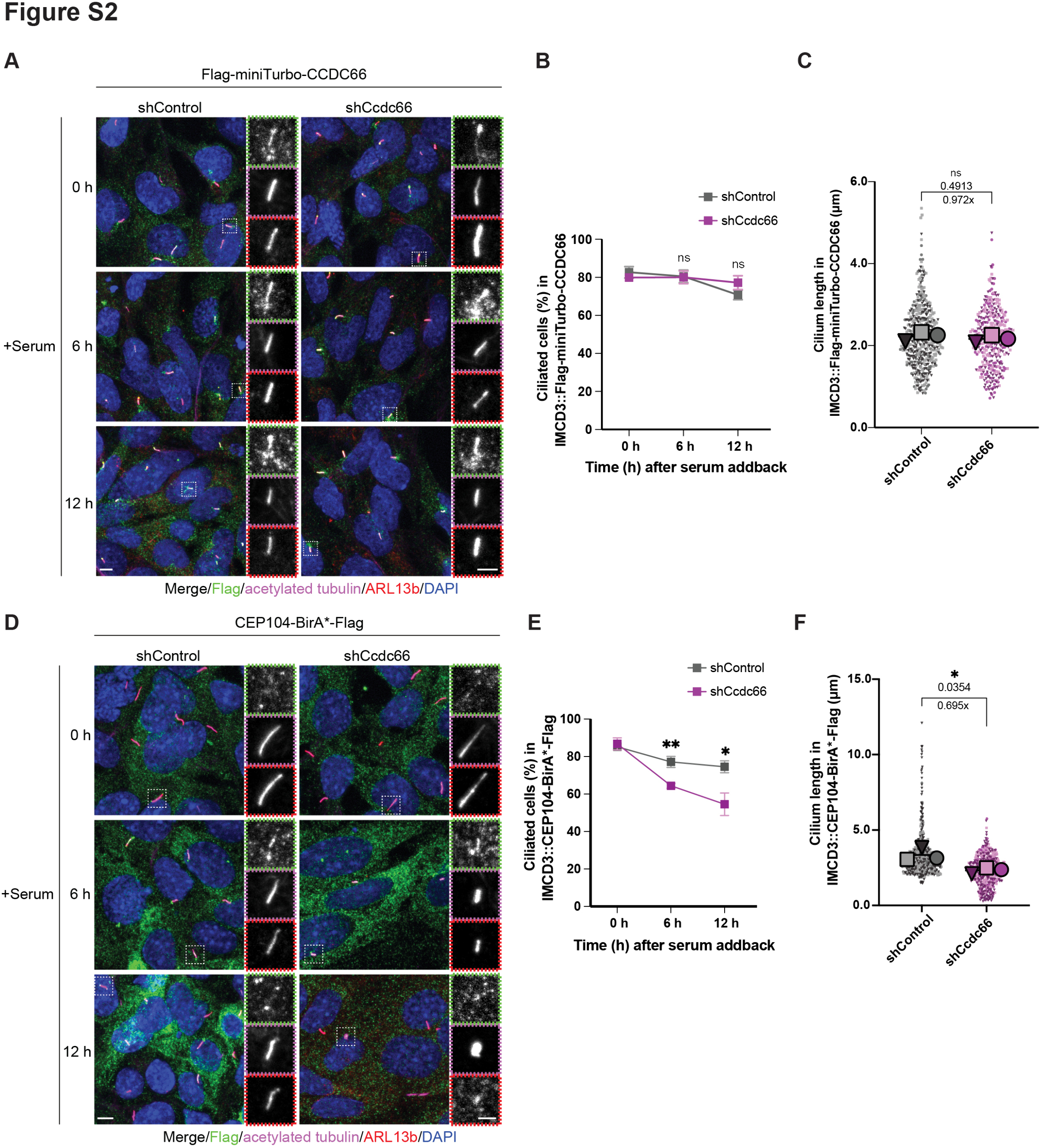

Supplement: S2 Fig — (A–C) IMCD3::Flag-miniTurboID- CCDC66 were transduced with control and CCDC66-targeting shRNA, selected and seeded on coverslips. After 48 h serum starvation, the cells were stimulated with serum-rich medium to induce cilia disassembly for total of 12 h, fixed and stained with anti-Flag, anti-acetylated-tubulin, anti-ARL13b and DAPI. Scale bar: 5 µm. Insets show 3× magnifications of the cilia, Scale bar: 2 µm (B) Quantification of cilia number in (A). Data represents mean ± SD of 3 independent experiments. n > 400 cells for control and CCDC66 depletion at all three indicated time points. Mean cilia percentage at 0 h is 82.68% for shControl, 79.87% for shCcdc66; at 6 h is 80.52% for shControl, 80.0% for shCcdc66; at 12 h is 70.75% for shControl, 77.22% for shCcdc66. (p values of multiple t tests of grouped data p = 0.2457, p = 0.8590, p = 0.0651, ns: not significant). (C) Quantification of cilia length in (A) at 0 h. Super plot represents individual experimental values with mean ± SEM of 3 independent experiments. n > 400 cells for each condition. Mean cilia length measured in 3D in CCDC66 depletion is decreased to 0.97-fold of the mean control length. (Welch t test p value ns: not significant). (D) IMCD3::CEP104-BirA*-Flag were transduced with control and CCDC66-targeting shRNA, selected and seeded on coverslips. After 48 h serum starvation, followed by serum addback for total of 12 h, fixed and stained with anti-Flag, anti-acetylated-tubulin, anti-ARL13b and DAPI. Scale bar: 5 µm. Insets show 3× magnifications of the cilia, Scale bar: 2 µm (E) Quantification of cilia number in (D). Data represents mean ± SD of 3 independent experiments. n > 400 cells for control and CCDC66 depletion at all three indicated time points. Mean cilia percentage at 0 h is 85.22% for shControl, 86.75% for shCcdc66; at 6 h is 77.13% for shControl, 64.41% for shCcdc66; at 12 h is 74.54% for shControl, 54.53% for shCcdc66. (p values of multiple t test of grouped data p = 0.5229, **p = 0.0084 [file pbio.3003313.s002.tif]

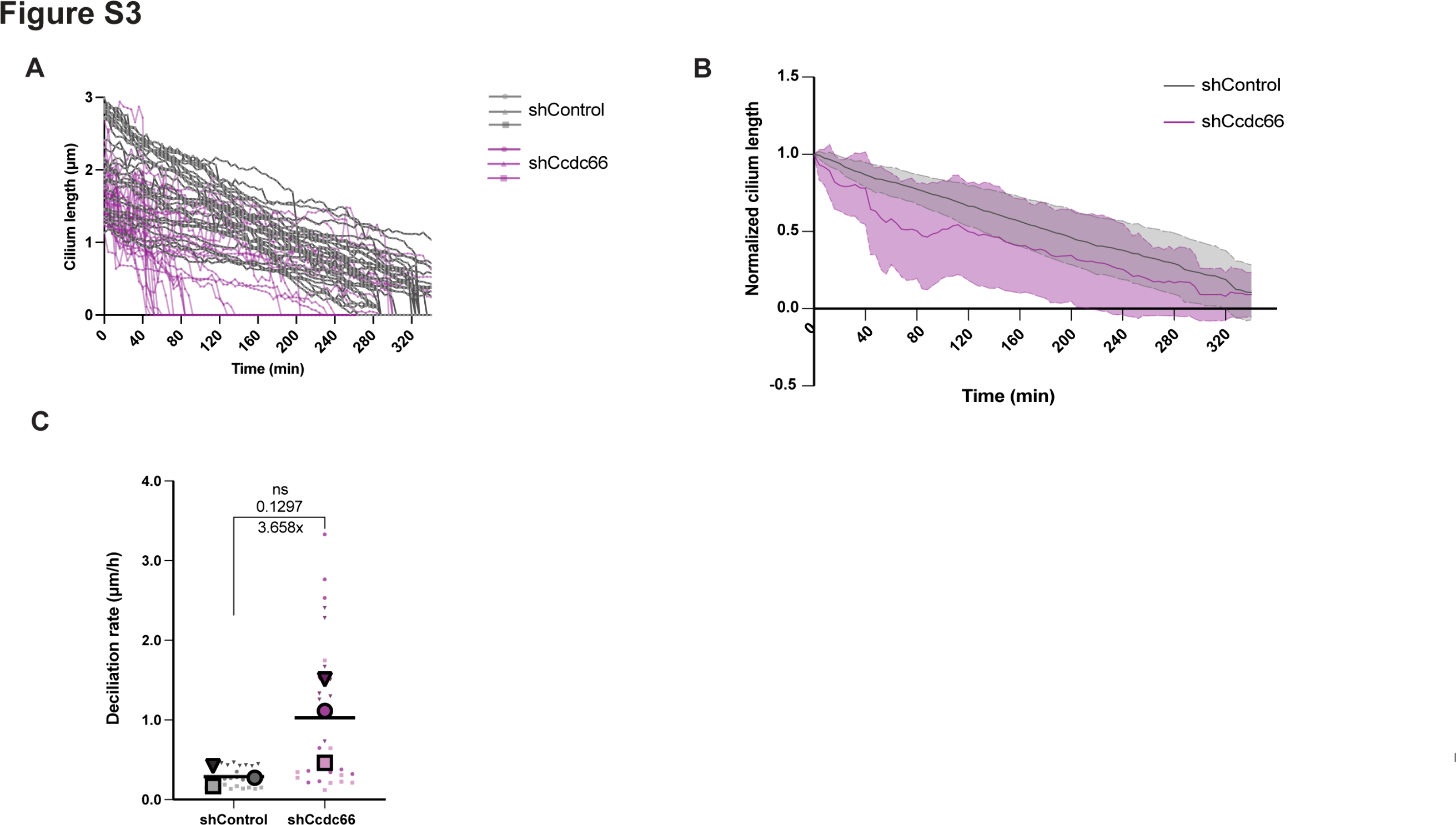

Supplement: S3 Fig — (A) Individual raw cilia length connecting line curves from Fig 3B, measured from fluorescence of three independent experiments. n = 30 cilia for both shControl and shCcdc66 conditions. (B) Normalized data of cilia length quantification in Fig 3B. Normalization is performed by dividing individual values with the average control value of experimental replicate at t = 0. (C) Deciliation rate of cilia from Fig 3B (the speed of cilium shortening calculated as the length lost over time), measured from fluorescence of three independent experiments and represented as the mean ± SD. n = 30 cilia total for both shControl and shCcdc66 conditions. The data underlying the graphs shown in the figure can be found in S1 Data. (TIF) [file pbio.3003313.s003.tif]

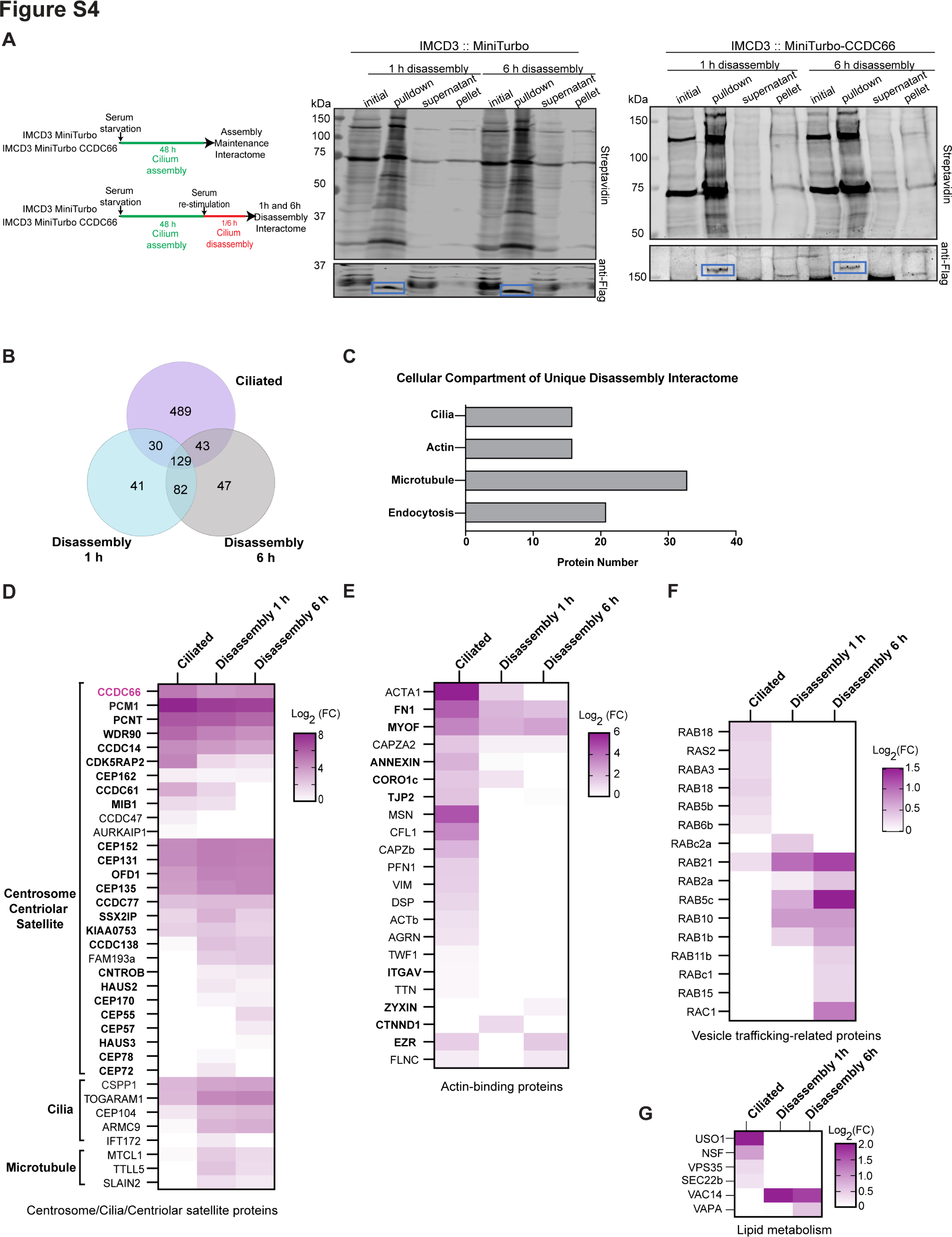

Supplement: S4 Fig — (A) Diagram depicting experimental plan of cell line treatments. Western blot analysis of samples from different stages of streptavidin pulldowns from IMCD3 cells expressing miniTurboID-Flag or miniTurboID-Flag-CCDC66. Cells were ciliated by serum starvation and then stimulated with serum for 1 h and 6 h. Samples were collected at the lysate (initial), pellet, and supernatant stages, and the non-eluted bead samples were run as the pulldown samples. Samples were blotted using Streptavidin-IRDye800 coupled and anti-Flag. Blue boxes show bands corresponding to Flag fusion proteins. (B) Comparison of the CCDC66 proximity interactomes of ciliation, 1 h disassembly and 6 h disassembly conditions. (C) GO‐enrichment analysis of the CCDC66 disassembly interactome based on their biological process and cellular compartment. The x‐axis represents the log‐transformed p‐value (Fisher’s exact test) of GO terms. (D–G) Heat map showing Log2(Fold Change) of the CCDC66 proximity interactors in ciliated cells versus 1 h and 6 h after serum stimulation of cilium disassembly. The range of the Log2 Fold Change (FC) values is from 0 to 8, represented by shades of purple. Categories were determined using DAVID functional annotation tool and literature mining. Centrosome/cilia/centriolar satellite proteins were plotted in (D) where proteins with both centrosome and centriolar satellite compartment association are shown in bold. Actin-binding proteins were plotted in (E) where proteins shown in bold are ciliary actin-related proteins. Proteins linked to vesicular trafficking and lipid metabolism were plotted in (F) and (G), respectively. The numerical data from the graphs shown in the figure can be found in S1 Data. GO, Gene Ontology. (TIF) [file pbio.3003313.s004.tif]

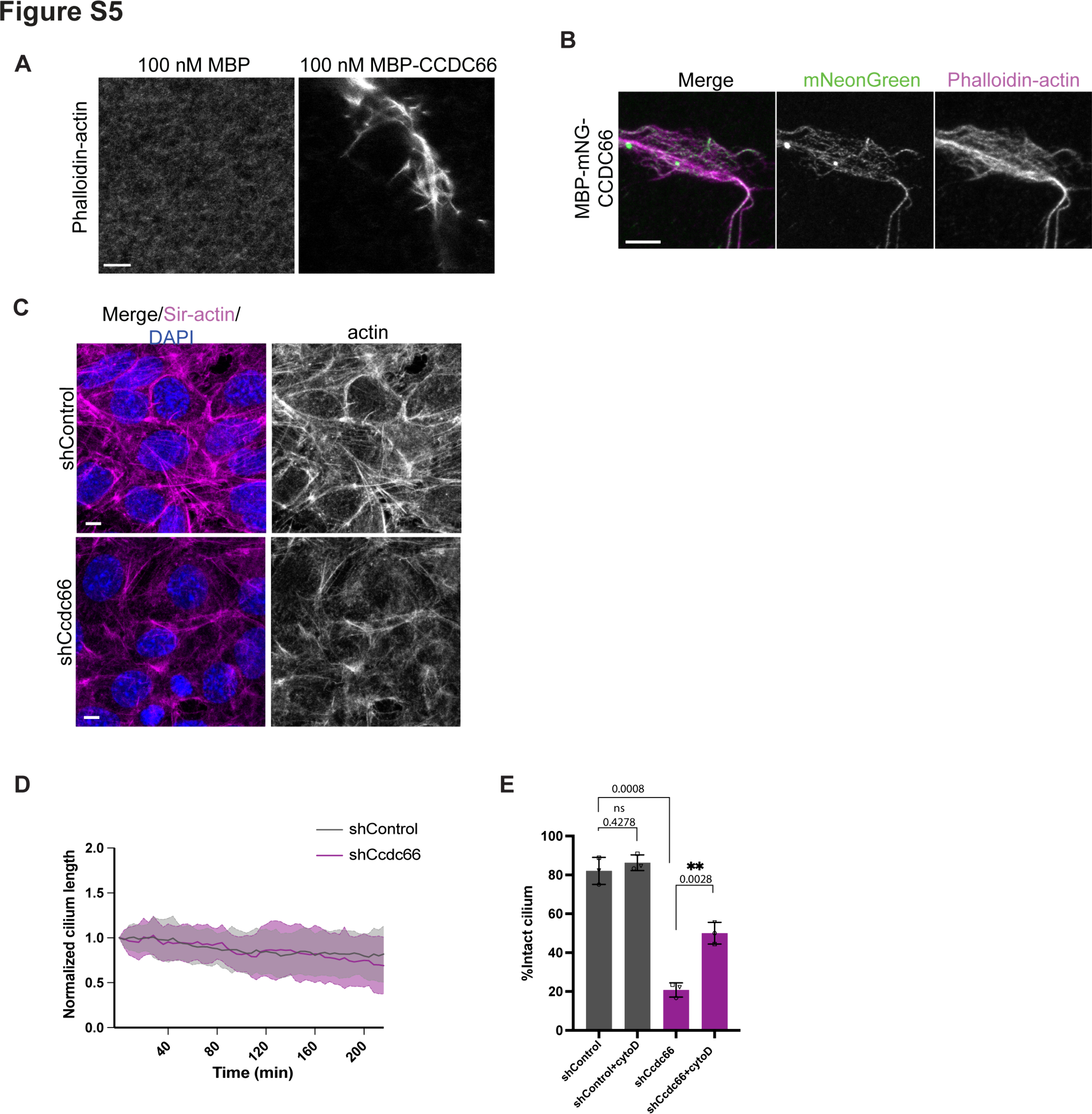

Supplement: S5 Fig — (A–B) CCDC66 binds and bundles actin filaments in vitro. MBP, MBP-CCDC66 or MBP-mNG and MBP-mNG-CCDC66 were mixed with Alexa 568 Phalloidin stabilized polymerized actin in polymerization buffer and the reaction mixtures were loaded into a flow chamber under a HCl-treated coverslip attached with double-sided tape to the microscope slide. Fluorescent proteins and actin were observed with a Leica SP8 confocal microscope. (C) Effects of CCDC66 depletion on actin cytoskeleton. IMCD3 cells transduced and stably expressing either control or shCcdc66 were grown on glass coverslips, fixed with 4% PFA and imaged with confocal microscopy. Cells were co-stained with Sir-Actin and DAPI. Scale bar 5 µm. (D) Normalized cilia length curve from Fig 6E for both shControl and shCcdc66 conditions. Normalization is performed by dividing individual values with the average control value of experimental replicate at t = 0. (E) Quantification of the percentage of intact cilia based on fluorescence from three independent experiments, represented as the mean ± SD. A total of n = 30 cilia from 3 independent experiments were analyzed for both shControl and shCcdc66 conditions, either in the absence or presence of CytoD. The data underlying the graphs shown in the figure can be found in S1 Data. (TIF) [file pbio.3003313.s005.tif]

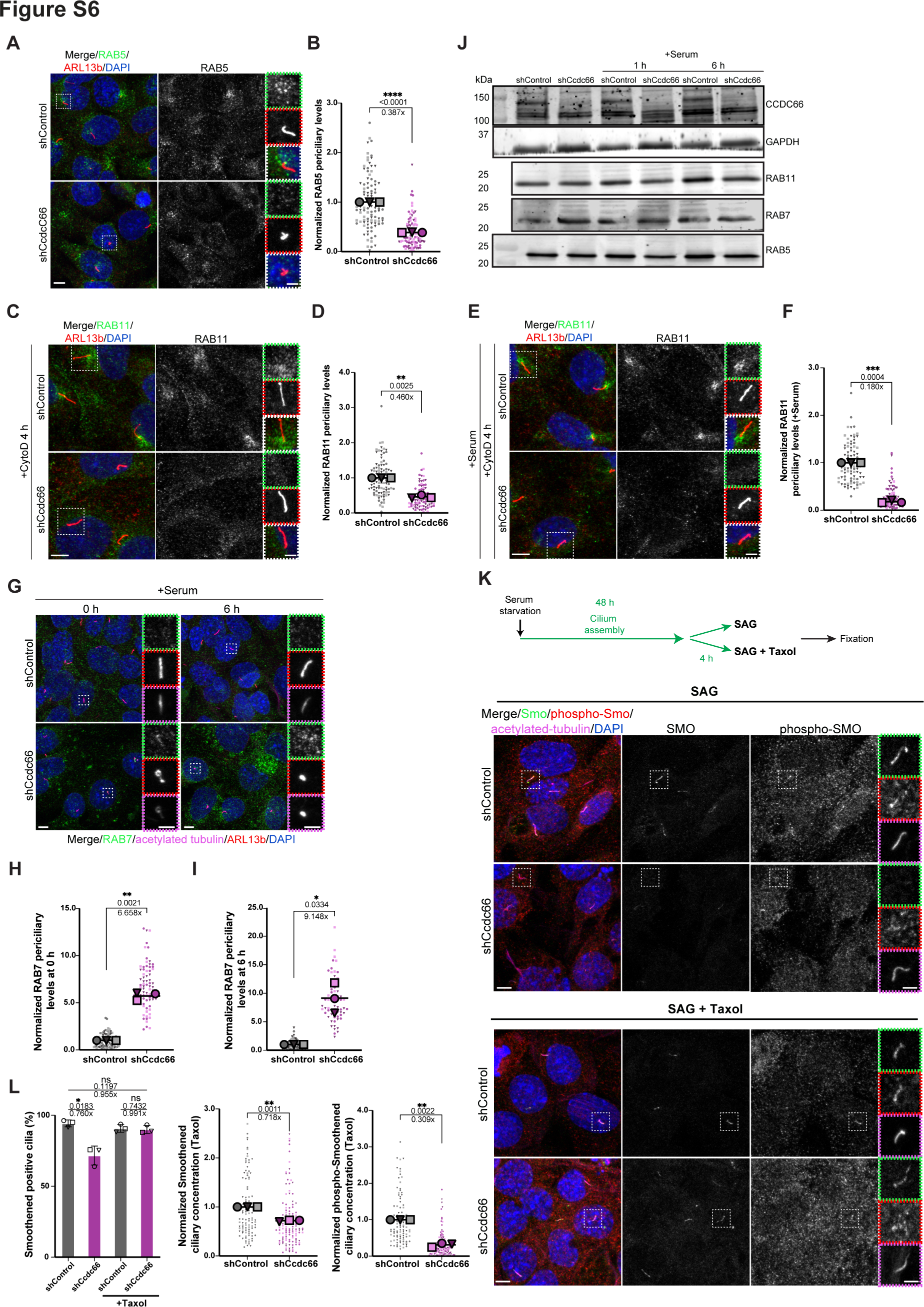

Supplement: S6 Fig — (A) 48 h-ciliated control and CCDC66-depleted cells were fixed with 4% PFA and stained against anti-RAB5, anti-ARL13b and DAPI. Scale bar 5 µm. Insets show 1.3× magnifications of the cilia, Scale bar: 2 µm. (B) Measured is the mean pixel intensity of a circular 5 μm2 area centered at the ciliary base of each cell in (A). Super plot of normalized individual experimental values with mean ± SEM represents 3 independent experiments. Normalization is performed by dividing individual values with the average control value of experimental replicate. The different colored experimental replicates are shown as either circle, squares or triangles and with different color lightness. Approximately 100 cells for each condition. Mean periciliary levels of RAB5 in CCDC66 depletion is decreased to 0.387-fold of the control mean. (Welch’s t test ****p < 0.0001). (C) 48 h-ciliated control and CCDC66-depleted cells were treated with 0.5 µM CytoD for 4 h then fixed with 4% PFA and stained against anti-RAB11, anti-ARL13b and DAPI. Scale bar 5 µm. Insets show 1.3× magnifications of the cilia, Scale bar: 2 µm. (D) Measured is the mean pixel intensity of a circular 5 μm2 area centered at the ciliary base of each cell in (C). Super plot of normalized individual experimental values with mean ± SEM represents 3 independent experiments. The different colored experimental replicates are shown as either circle, squares or triangles and with different color lightness. Approximately 100 cells for each condition. Mean periciliary levels of RAB11 in CCDC66 depletion is 0.46-fold of the control mean. (Welch’s t test **p = 0.0025). (E) 48 h-ciliated control and CCDC66-depleted cells were serum stimulated and simultaneously treated with 0.5 µM CytoD for 4 h then fixed with 4% PFA and stained against anti-RAB11, anti-ARL13b and DAPI. Scale bar 5 µm. Insets show 1.3× magnifications of the cilia, Scale bar: 2 µm. (F) Measured is the mean pixel intensity of a circular 5 μm2 area centered at the ciliary base [file pbio.3003313.s006.tif]

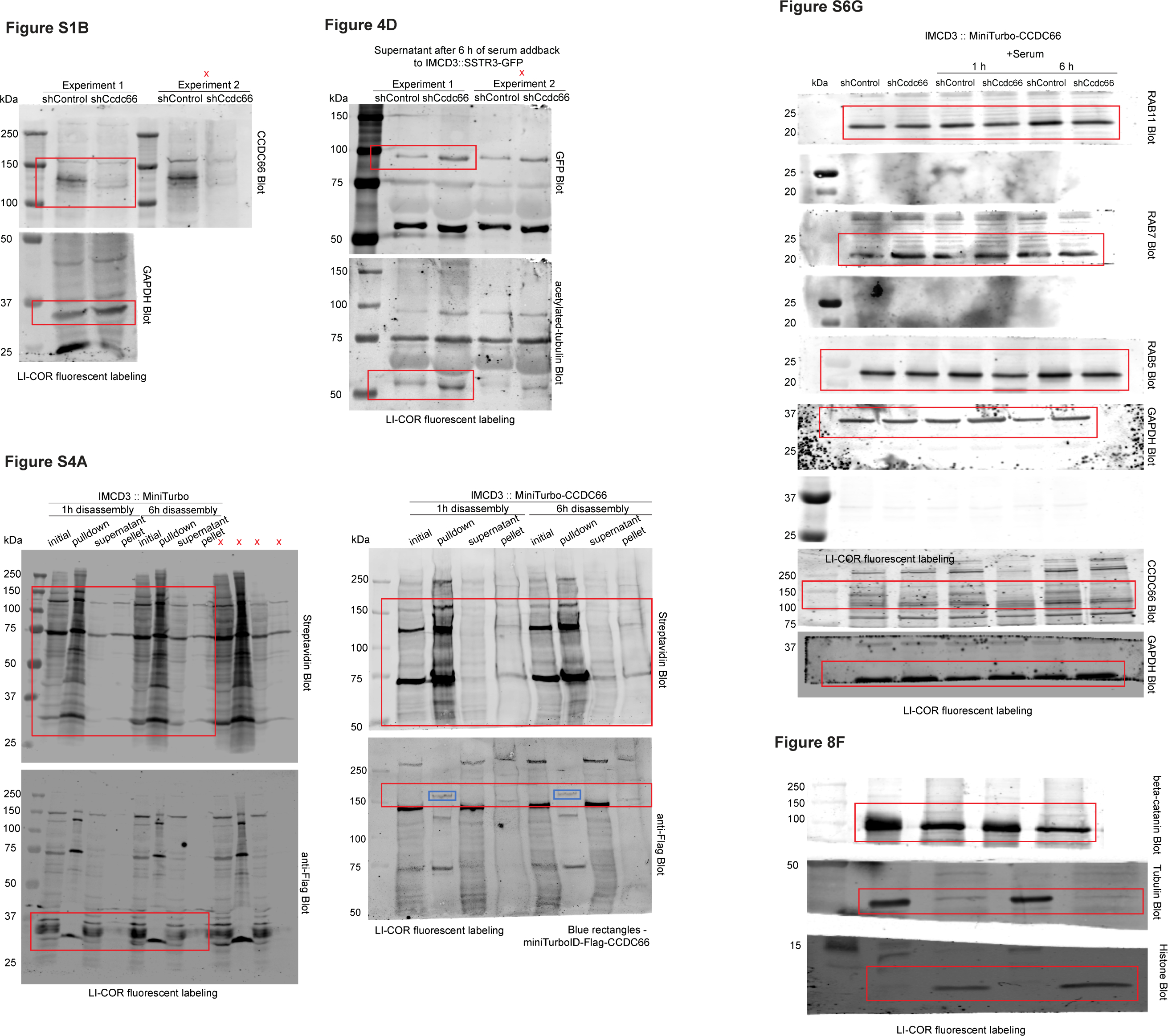

Supplement: S1 Raw Images — Red boxes are used to show the represented blot in the figures. Red crosses show the parts of the blot that are not used in the figures. (TIF) [file pbio.3003313.s010.tif]
