# Supplementary material for: CCDC66 regulation of cytoskeleton and cilia stability is important for signaling and epithelial organization
Source: PLoS Biol. 2025 Jul 29;23(7):e3003313. doi: 10.1371/journal.pbio.3003313 (PMC12324684; doi:10.1371/journal.pbio.3003313)
Supplement: Attachment — Submitted filename: ResponseToReviewer_15May_final.pdf [file pbio.3003313.s013.pdf]

Response to Reviewers

We thank the reviewer for his/her comments and constructive suggestions. Here, we explain the revisions, additional experimentations and analyses we performed to address the points raised by the reviewer. For reference, the reviewer comments are shown in blue and our responses in black.

Reviewer#2

The paper by Deretic et al. on "CCDC66 regulation of cytoskeleton and cilia stability is important for signaling and epithelial organization" has been really improved by the major revisions done. The authors respond to all my previous comments. However, the substantial changes done to the paper raised a few new issues that should be addressed prior to publication.

We thank the reviewer for acknowledging our substantial revisions and for offering additional constructive feedback that will strengthen the conclusions from the revision experiments and further improve the clarity of the manuscript.

Notably, some experiments were done on assembling/steady state cilia while others were done on disassembling cilia. While it might be that both processes use the same mechanisms to control ciliary length, it makes the paper difficult to follow. Putting both processes on the same figures to compare (including in the model) may help. There is also seemingly contradictory results that make the understanding very hard and are not discussed.

We agree that a clearer separation of cilium-maintenance and cilium-disassembly experiments improves the manuscript's readability. We have addressed the reviewer's concerns in two ways:

1. **Re-organization of figures:** We consolidated all experiments examining CCDC66's role in cilium maintenance/steady-state into Figures 1–2 and those analyzing cilium disassembly into Figures 3–5. The data evaluating the downstream consequences of CCDC66 loss, regulation of ciliary content, trafficking, signaling, and epithelial organization, remain at the end of the manuscript, as these phenotypes likely reflect both ciliary and non-ciliary functions of CCDC66.
2. **Updated schematic model (Figure 7).** We added the model figure to illustrate how CCDC66-mediated regulation of microtubule stability, ectocytosis, and Rab-mediated trafficking might operate during both cilium maintenance and disassembly. As the reviewer noted, our data support these as common mechanisms underlying these two ciliary phases. To highlight this, we revised the legend accordingly.

My understanding of the authors hypothesis is that during cilium assembly and maintenance, primary cilia underwent few events of ectocytosis (mainly EVs) while microtubules are stable. Events of ectocytosis may dependent on the actin cytoskeleton. In absence of CCDC66, ectocytosis is increased and microtubule destabilized leading to their fragmentation. Thus, at this stage like during disassembly, cytoD should at least partially rescue ciliary length and decrease ectocytosis both in control and Ccdc66 depleted cells. Taxol should lead to primary cilia of the same size in control and depleted cells, maybe slightly smaller than in none treated cells due to the decrease availability of free tubulin. Could the authors check whether it works according to their model?

We agree with the reviewer's summary of our working model. Based on our data, we propose that that actin-dependent ectocytosis, Rab-mediated trafficking, and microtubule stability act

together to regulate cilium integrity, thereby governing both cilium maintenance and disassembly.

In response to the reviewer's request, we measured cilium length during maintenance after CytoD or Taxol treatment. The results, summarized below, further support a shared mechanism for maintenance and disassembly.

1. Effects of Taxol treatment on steady-state cilia
Revision Figure 1 largely aligns with this reviewer's expectations regarding the effects of Taxol. Consistent with prior studies, treatment with Taxol (1 μM for 4 hours) induced disassembly of cilia in control cells, with a reduction of 22%, likely due to the overstabilization of microtubules and decreased soluble tubulin levels in the cytoplasm of control cells[1]. In contrast, there was no change in ciliation in CCDC66-depleted cells, suggesting that Taxol restores the microtubule destabilization caused by CCDC66 depletion. Similarly, Taxol significantly reduced cilia length in control cells. Interestingly, in CCDC66-depleted cells, Taxol resulted in a marginal increase in cilia length, which were also somewhat larger than the cilia in control cells.

These results indicate that the stabilization of cytoplasmic and axonemal microtubules by Taxol has a positive impact and partially rescue ciliary phenotypes in CCDC66-depleted cells. A similar effect of Taxol was noted during cilium disassembly (as shown in Fig. 3D-F); however, it did not elevate ciliary length in CCDC66-depleted cells to maintenance levels, likely due to the influence of other microtubule-related destabilization signals during disassembly.

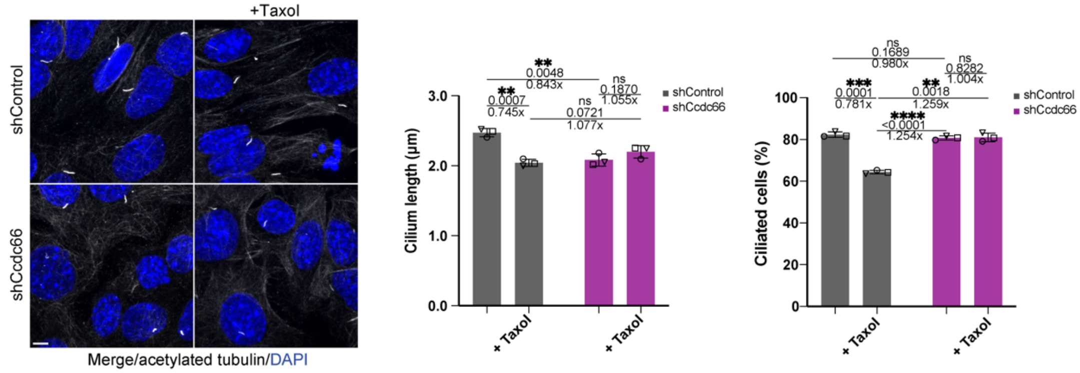

3. **Effects of CytoD treatment on steady-state cilia**
Revision Figure 2 indicates that treatment with CytoD (0.5 μM for 4 hours) positively affected ciliary length in both control and CCDC66-depleted cells, with a more pronounced effect in CCDC66-depleted cells. Therefore, we can conclude that a low dose of CytoD effectively rescued the shortened steady-state cilia phenotype observed in CCDC66-depleted cells. Additionally, CytoD treatment increased the percentage of ciliated cells, aligning with previously reported roles of actin inhibition in promoting ciliogenesis. Overall, these results suggest that CytoD has a slightly greater impact on cilium maintenance in CCDC66-depleted cells (see Revision Figure 2, Fig. 2) compared to disassembly (Fig. 6A, 6C). This difference is likely due to distinct environmental and cellular signals that regulate actin during cell cycle re-entry, as well as microtubule and RABs contribution to cilia disassembly.

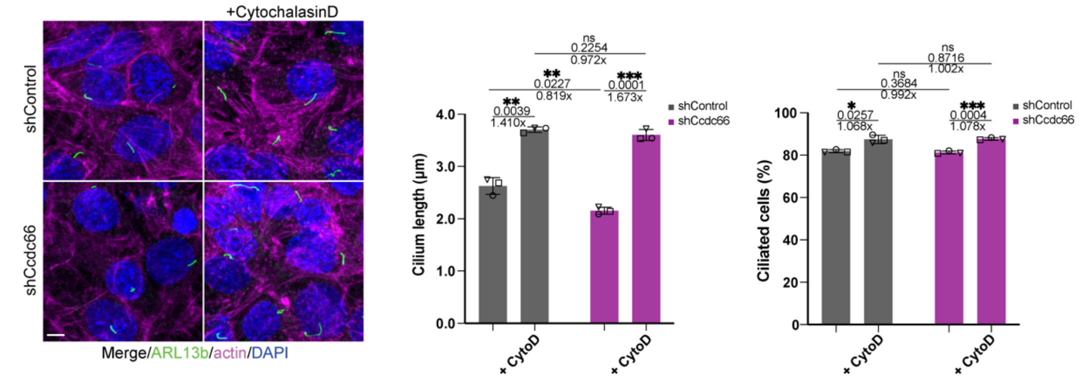

Since we observed similar effects of Taxol and CytoD on steady-state cilia compared to disassembling cilia, we decided to include this data only in the rebuttal letter to avoid introducing additional complexity and confusion related to analyzing multiple ciliary states, as highlighted by the reviewer in Comment #1.

Intriguingly, CCDC66 may interact and bundle actin (difficult to say without the control image of the actin network in vitro in figure S5A). Accordingly, its depletion seems to decrease cytoplasmic actin cables. Therefore, we would expect less actin in the primary cilia and thus less ectocytosis in depleted cells, the reverse of what is observed. How do the authors reconcile these paradoxical results? Could it be that depletion of Ccdc66 leads to a decrease accumulation of actin at the tip and more shedding events all along the primary cilia?

Using the flow chamber, visualizing the thin and fast-moving actin filaments was challenging, as these typically require TIRF-based imaging of stabilized and surface-attached actin. We have now included a control image containing actin and MBP-CCDC66, where we could only detect a faint actin filament network (Fig. S5A). In comparison to this control, it is evident that CCDC66 interacts with and bundles actin filaments *in vitro*.

As the reviewer correctly pointed out, however, depletion of CCDC66 results in fewer cytoplasmic actin filaments (Fig. S5C) but increased ectocytosis events (Fig. 4C, 4D), a phenotype indicative of increased actin polymerization.

We propose that this discrepancy arises from the context-dependent roles of CCDC66, potentially forming distinct protein complexes in different cellular compartments. Additionally, actin filament regulation varies significantly depending on subcellular localization and functional context such as cytoplasmic stress fibers, branched cortical actin, cilium tip-associated actin, or axoneme-associated actin. Therefore, attributing the cellular phenotype directly to the *in vitro* bundling activity of CCDC66 would oversimplify the complexity of actin dynamics in cells. We suggest that the differences between our *in vitro* and cellular observations stem primarily from the diverse regulatory mechanisms controlling actin dynamics and from distinct interaction partners of CCDC66 in different cellular compartments. It is, therefore, not excluded that depletion of CCDC66 leads to a decrease in actin accumulation at the tip and more shedding

events all along the primary cilia. This is a very attractive hypothesis which would be interesting to dissect with electron microscopy in the future.

We have extended this interpretation from the Discussion section in pg. 25 as follows:

….. CCDC66's ciliary functions are partially mediated by the processes involving actin, such as ciliary ectocytosis. We note that actin-mediated regulation of the cilium is multifaceted, involving different pools: the ciliary pool for ectocytosis, the cytoplasmic pool for myosin VI-dependent transport of ciliary proteins, and cortical actin for length regulation [1-4]. These findings imply that CCDC66 may regulate ectocytosis, actin-mediated trafficking, or cortical actin by modulating actin dynamics during cilium disassembly and length regulation. Additionally, given its direct affinity for both actin and microtubules, CCDC66 may regulate the ciliary cytoskeleton and related events by crosslinking microtubules with actin [5,6]. While dissecting the specific functions of the various cytoskeletal pools of CCDC66 will be challenging, future *in vitro* studies are expected to provide crucial insights into how CCDC66 regulates microtubule and actin dynamics.

During primary cilia disassembly, disassembly and cilia shortening depends on the actin cytoskeleton. However, ectocytosis seems not to be affected in control by Cytochalasin D treatment. Thus, what could be the role of actin? Disassembly but not cilia shortening (isn't it paradoxical?) also depends on microtubule stability through HDAC6/Aurora A. In absence of CCDC66, there is more events of cilia fragmentation that depends at least partially of the actin cytoskeleton and maybe of microtubule destabilization.

As noted by the reviewer, our data indicate that both actin and microtubules contribute to CCDC66-mediated regulation of ciliary events during disassembly. To define the roles of actin, we examined the effects of CytoD treatment on different ciliary phenotypes. We quantified ectocytosis events and defined “intact cilia” as those lacking vesicle or fragment ectocytosis during imaging. While CytoD treatment modestly increased intact cilia in control cells (from 80% to 85%), it significantly improved ciliary stability in CCDC66-depleted cells (from 20% to 45%) (Fig. S5E, 6F). CytoD treatment also effectively reversed other defects induced by CCDC66 depletion, including reduced cilium shortening, decreased length fluctuations, and inhibited disassembly (Fig. 6C–H, S5E–F). These findings highlight the importance of actin-dependent mechanisms underlying CCDC66's role in regulating cilia.

However, we acknowledge that CytoD effects may involve actin populations beyond intraciliary actin, such as cytoplasmic or cortical networks, or actin involved in trafficking and signaling pathways. Moreover, as the reviewer emphasized and supported by our data, microtubules also regulate these events, and the crosstalk between actin and microtubule dynamics during ciliary remodeling remains poorly understood. Given our incomplete understanding of these regulatory mechanisms, we have refrained from overinterpreting our data. We have now clearly highlighted these complexities in the revised Discussion and emphasized the need for further investigation to better elucidate how distinct actin pools coordinate with microtubule dynamics during cilium remodeling.

Minor points:

* There are a few inaccuracies that should be removed:

- Primary cilia are not only assembled in cell exiting the cell cycle, but observed in most cells in G1.
We corrected this.

- A cell entering mitosis cannot be quiescent.
We corrected this.

- On page 7 the results on Cep164, Talpid3 and CP110 are described two times.
We thank the reviewer for raising this point. These data represent the only dataset in our manuscript performed with two replicates. Since the results were negative and peripheral to the primary focus of this study, we did not perform additional replicates for the revision.
In Fig. 1A, we showed that depletion of CCDC66 in IMCD3 cells affects cilium length but not ciliation efficiency. Therefore, the absence of defects in the recruitment of ciliogenesis regulators to the basal body upon CCDC66 depletion was expected. In the revised manuscript, we have removed this quantification from the supplementary figures. This revision clarifies our manuscript's focus and effectively differentiates this study from our previous work, which specifically addressed the role of CCDC66 during cilium assembly. In that earlier study, we showed that CCDC66 depletion in RPE1 cells impaired cilium assembly by affecting the recruitment of ciliogenesis factors to centrosomes and cilia. We have clearly discussed this prior work and the distinct phenotype observed in RPE1 cells within our manuscript.

- It is not always clear whether statistics were applied on the three repeats of an experiment or on all the values measured in these three experiments. Please clarify.
We performed statistical analyses using the means obtained from three experimental replicates. To clarify this, we carefully reviewed and updated all figure legends to ensure each graph clearly explains the statistical methods used. Additionally, we expanded the Methods section to provide more detailed descriptions of our statistical procedures.

- There is still experiments with only two repeats in the supplementary data.
As we addressed above, the data in Fig. S1 represented the only dataset in our manuscript performed with two replicates. Because these results on assembly were negative and peripheral to the primary focus of our study on maintenance and disassembly, we did not perform additional replicates during revision. To enhance clarity and maintain focus, we have now removed this data set from the revised manuscript.

To ensure that all data supporting our conclusions are quantified, we included measurements of the percentage of SMO-positive cilia and the ciliary levels of SMO and phospho-SMO in control and CCDC66-depleted cells treated with taxol (Fig. S6K, S6L).

- There is some inaccuracies on the figure numbering in the text.
We carefully checked the manuscript and corrected all inaccuracies in figure numbering.

- Cytochalasin D affects both branched and linear actin filaments.
CytoD was shown to inhibit actin polymerization primarily by binding to the barbed ends of linear actin filaments, preventing further monomer addition [7].Therefore, it does not directly distinguish between linear and branched actin filaments; instead, it affects actin dynamics broadly by capping growing filament ends. Notably, the cellular effects of CytoD, including its

impact on ciliogenesis, vary depending on concentration, exposure duration, and cell type. In our experiments, we used a low dose of CytoD (0.5 μM), previously demonstrated to promote ciliogenesis, increase cilium length, and inhibit cilium disassembly, but did not affect the distribution of the more stable F-actin at cell-cell junctions and the basal stress fibers [8-11](. This is confirmed with our staining of residual actin filaments after CytoD treatment (see Revision Figure 2).

- It would help to mention in the figure legend against what were normalized quantifications.
We have included details on normalization clearly in the figure legends. Data was normalized to the average value of the control cells.

- There is a problem with the legend of at least Figure 6 and S5
We revised and corrected the legends for Figures 6 and S5.

* What means intact cilia?
"Intact cilia" refers to cilia that do not undergo vesicle ectocytosis or fragmentation during the imaging period. We have clarified this definition in both the figure legends and the manuscript text.

* Over interpretations should be avoided. E.g., The paper do not demonstrate that CCDC66 plays a role in activating HH and Wnt by regulating intraciliary and vesicular transport.
We have moderated our conclusions throughout the manuscript to avoid overinterpretation.

References

1 Sharma, N., Kosan, Z. A., Stallworth, J. E., Berbari, N. F. & Yoder, B. K. Soluble levels of cytosolic tubulin regulate ciliary length control. *Mol Biol Cell* **22**, 806-816 (2011). https://doi.org/10.1091/mbc.E10-03-0269

2 Brucker, L., Kretschmer, V. & May-Simera, H. L. The entangled relationship between cilia and actin. *Int J Biochem Cell Biol* **129**, 105877 (2020). https://doi.org/10.1016/j.biocel.2020.105877

3 Smith, C. E. L., Lake, A. V. R. & Johnson, C. A. Primary Cilia, Ciliogenesis and the Actin Cytoskeleton: A Little Less Resorption, A Little More Actin Please. *Front Cell Dev Biol* **8**, 622822 (2020). https://doi.org/10.3389/fcell.2020.622822

4 Wang, S. *et al.* Functions of actin-binding proteins in cilia structure remodeling and signaling. *Biol Cell* **115**, e202300026 (2023). https://doi.org/10.1111/boc.202300026

5 Batman, U., Deretic, J. & Firat-Karalar, E. N. The ciliopathy protein CCDC66 controls mitotic progression and cytokinesis by promoting microtubule nucleation and organization. *PLoS Biol* **20**, e3001708 (2022). https://doi.org/10.1371/journal.pbio.3001708

6 Jijumon, A. S. *et al.* Lysate-based pipeline to characterize microtubule-associated proteins uncovers unique microtubule behaviours. *Nat Cell Biol* **24**, 253-267 (2022). https://doi.org/10.1038/s41556-021-00825-4

7 Wakatsuki, T., Schwab, B., Thompson, N. C. & Elson, E. L. Effects of cytochalasin D and latrunculin B on mechanical properties of cells. *J Cell Sci* **114**, 1025-1036 (2001). https://doi.org/10.1242/jcs.114.5.1025

8 Hufft-Martinez, B. M., Wang, H. H., Saadi, I. & Tran, P. V. Actin cytoskeletal regulation of ciliogenesis in development and disease. *Dev Dyn* **253**, 1076-1093 (2024). https://doi.org/10.1002/dvdy.724

9 Kim, J. *et al.* Actin remodelling factors control ciliogenesis by regulating YAP/TAZ activity and vesicle trafficking. *Nat Commun* **6**, 6781 (2015). https://doi.org/10.1038/ncomms7781

10 Li, A. *et al.* Ciliary transition zone activation of phosphorylated Tctex-1 controls ciliary resorption, S-phase entry and fate of neural progenitors. *Nat Cell Biol* **13**, 402-411 (2011). https://doi.org/10.1038/ncb2218

11 Phua, S. C. *et al.* Dynamic Remodeling of Membrane Composition Drives Cell Cycle through Primary Cilia Excision. *Cell* **168**, 264-279 e215 (2017). https://doi.org/10.1016/j.cell.2016.12.032
