# Supplementary material for: CCDC66 regulation of cytoskeleton and cilia stability is important for signaling and epithelial organization
Source: PLoS Biol. 2025 Jul 29;23(7):e3003313. doi: 10.1371/journal.pbio.3003313 (PMC12324684; doi:10.1371/journal.pbio.3003313)
Supplement: Attachment — Submitted filename: ResponseToReviewers_final.docx [file pbio.3003313.s011.docx]

**Response to Reviewers**

We thank the reviewers for their comments and constructive suggestions to improve our manuscript. Here, we explain the revisions, additional experimentations and analyses we performed to address the points raised by the reviewers. For reference, the reviewer comments are shown in blue and our responses in black.

**Reviewer#1**
Deretic et al claimed that Ccdc66 is a microtubule stabilizing factor in epithelial tissues and is essential for primary cilium maintenance and disassembly. In this manuscript, the authors observed the effect of Ccdc66 depletion on each process of cilia assembly, maintenance, and disassembly in IMCD3 cell line using several molecular/genetic tools. Additionally, the authors sought to demonstrate the essential role of Ccdc66-mediated cilia in epithelial organization through the control of Shh/Wnt signaling. Although these observations, and particularly the cilia disassembly-specific data, are interesting, the logic and evidence for claiming a role for Ccdc66 limited to cilia maintenance/degradation is somewhat weak. Moreover, some experimental conditions are inadequate to verify their hypotheses. Listed below are several main concerns that must be addressed.

We thank the reviewer for the summary of our results and constructive feedback. The perceived weakness in the logic for CCDC66’s functions may be due to how we initially presented our questions, approaches and interpreted our conclusions. To clarify our findings and their impact, we have revised the flow of our manuscript and included a graphical model (Fig. 9). Additionally, in response to suggestions from all reviewers, we have made substantial revisions to the manuscript and performed many new experiments to address any ambiguities and strengthen our conclusions.

Here is a summary of the new structure of our manuscript:

1. CCDC66 is a MAP that localizes to the axoneme of the primary cilium.
2. CCDC66 is required for maintaining cilium length and stability in both steady-state and disassembling primary cilia, relying on its microtubule-stabilizing activity.
3. The CCDC66 proximity interactome during cilium disassembly reveals new relationships, which we further characterized:
   - Actin cytoskeleton dynamics contribute to CCDC66’s roles in regulating cilium length and stability, partly through regulating ciliary exocytosis.
   - CCDC66 influences vesicular trafficking via specific RAB proteins at the ciliary base.
4. CCDC66 is required for regulating cilium composition, ciliary transport, and Wnt/Hedgehog signaling.
5. CCDC66 is required for cellular signaling and epithelial cell organization through its ciliary and non-ciliary functions.

Finally, to better illustrate our findings, we revised the title of our manuscript to “**CCDC66 regulation of cytoskeleton and cilia stability is important for signaling and epithelial organization”**

**Major comments:**
1. I do not understand the condition inducing cilia assembly in the IMCD3 cell line utilized in this study. The authors stated that RPE1 cells assemble cilia via the intracellular pathway, whereas IMCD3 cells assemble cilia via the extracellular pathway. However, they induced ciliogenesis by serum starvation in IMCD3 as well as in RPE1 cells. Is there any evidence that these two different cell types respond/utilize intracellular or extracellular pathways differently under the same conditions, such as serum starvation?

We apologize for any lack of clarity. The pathways for ciliogenesis, whether intracellular or extracellular, are intrinsic to specific cell types and are not dependent on the method of ciliogenesis induction, such as serum starvation. IMCD3 cells, which are polarized kidney epithelial cells, utilize the extracellular pathway, while RPE1 cells employ the intracellular pathway to assemble cilia. This knowledge and its supporting evidence are detailed in several excellent papers and reviews on this topic, including some already cited in our manuscript [1-4]. The literature also highlights differences in the architectural organization of cilia between IMCD3 and RPE1 cells, such as the presence of a membrane ciliary pocket [5]. Additionally, there is evidence suggesting that different molecular players, such as Rab34, are involved in distinct ciliogenesis pathways [6].

2. Based on the authors' data, it may be right that Ccdc66 may not be essential for initiating ciliary assembly. However, it is clear that depletion of Ccdc66 in serum-starved IMCD3 cells results in shorter cilia. Therefore, the data may be interpreted as Ccdc66 deficiency resulting in incomplete ciliary assembly with abnormal ciliary length rather than promoting ciliary disassembly affecting microtubule stability. More concerning is that the authors are considering cells with short cilia as normally ciliated cells. Many studies still report that short cilia are a cilia assembly defect, thus the authors need to clarify the role of Ccdc66 for cilia assembly in IMCD3 cells.

We agree with the reviewer that we should have more clearly specified at which stage of the cilium assembly pathway was affected upon CCDC66 depletion. We do not consider short cilia to be normal; instead, our data suggest that short cilia in CCDC66-depleted cells may result from defects in axoneme elongation and stability during cilium assembly. Data in Fig. 1 show that CCDC66-depleted and control cells ciliate with similar efficiency, and Fig. S1 demonstrates that CCDC66-depleted cells do not exhibit defects in the recruitment of early ciliogenesis factors to the basal body or in the removal of the centriole cap. Together with the short cilia phenotype, this indicates that CCDC66 is not required for the initiation of cilium assembly but is essential for assembling cilia with proper length.

In summary, we agree with the reviewer's suggestion that CCDC66 plays a role in cilium assembly. To clarify these points, we removed all instances of "CCDC66 is not required for assembly" from the manuscript. We revised these sections to specify that while CCDC66 is not necessary for the initiation of cilium assembly, it is essential for axoneme elongation and stability during this process.

Regarding our conclusions on the role of CCDC66 in cilium disassembly by regulating microtubule stability, we differentiate this from assembly because disassembly is induced by serum stimulation in ciliated cells. However, we emphasize that similar mechanisms, specifically microtubule stabilization and ciliary ectocytosis, might contribute to defects during cilium assembly, maintenance, and disassembly.

3. In the EV analysis, I do not understand why the authors compared EVs between control cells under serum starvation and Ccdc66-depleted cells under serum addition. To distinguish that EVs from Ccdc66-depleted cells originated from cilia, it would be more feasible for the authors to compare EVs between control and Ccdc66-deficient cells under conditions of ciliary disassembly, i.e., serum addition.

We want to clarify that, as suggested by the reviewer, we compared extracellular vesicles (EVs) from control and CCDC66-depleted cells under identical conditions, following 6 hours of induced cilium disassembly. For these experiments, both groups were first ciliated for 48 hours, followed by the addition of serum for 6 hours to initiate cilia disassembly. Afterward, the supernatants were analyzed by western blotting. This might not have been clear enough from the sentence on page 14: “*In a complementary analysis, we purified extracellular vesicles (EVs) from the supernatants of serum-starved control and Ccdc66-depleted IMCD3::GFP-SSTR3 cells 6 h after serum stimulation.”*

We revised this sentence to: “*In a complementary analysis, we purified extracellular vesicles (EVs) from the supernatants of control and CCDC66-depleted IMCD3::GFP-SSTR3 cells. These cells were first serum-starved, then subjected to 6 hours of serum stimulation to induce cilia disassembly.”*

For further clarification, we have included a detailed experimental workflow in (Fig. 4D).

4. In fig.5 experiments, why did the authors compare data from the ciliogenesis proteome of control cells to the ciliary disassembly interactome of Ccdc66-deficient cells? In addition, the authors should compare their data directly with proteomes changed under the same condition, i.e., cilia disassembly condition following serum addition of IMCD3, instead of known ciliary disassembly proteome data sets.

We would like to clarify our experimental design and analysis methods, which are detailed in the results and methods section of our manuscript. We used cells stably expressing miniTurboID (control biotinylation) and miniTurboID-CCDC66 to identify proximity interactors of CCDC66 during cilium disassembly.

In these experiments, we did not deplete any proteins from the cells. Instead, we serum-starved miniTurbo or miniTurbo-CCDC66-expressing cells for 48 h to induce cilia formation, then initiated cilium disassembly by serum stimulation for 1 and 6 hours, along with 30 minutes of biotin labeling. We performed mass spectrometry and NSAF analysis to define the high-confidence CCDC66 disassembly proximity interactome. We then compared this disassembly interactome with that of cells possessing steady-state cilia (Fig. 5). This comparison enabled us to explore how the interactome is rewired during cilium disassembly.

For further clarification, we have included a detailed experimental workflow in (Fig. S4A).

5. In this study, the authors mainly have claimed that Ccdc66 has little effect on the initiation of cilia assembly in IMCD cells. However, as shown in Fig.6, in cells depleted of Ccdc66, both Shh and Wnt signaling, which are known to be essential signals for initial cilia assembly, were affected. How do the authors explain this kind of discrepancy about the role of Ccdc66 on the cilia assembly mechanism in IMCD?

Based on the literature regarding these signaling pathways and their connection to the primary cilium, we do not believe there is a discrepancy concerning the role of CCDC66 in cilium assembly. Below, we clarify the evidence supporting our conclusion. Moreover, we have incorporated key insights regarding the link between Hedgehog, Wnt, and primary cilia into the introduction, results, and discussion sections, as appropriate (pgs. 3, 27, 28, 29, 31, 32, 33, 38, 39, 40)

**Primary cilium and Hedgehog signaling:**

Cilia can assemble independently of the Hedgehog (Hh) signaling pathway but are crucial for transducing it [7]. Upon Hh stimulation, Smo activates and accumulates in the cilium, leading to the transcription of Hh target genes. Over a decade of research has shown that disruptions in cilia structure affect Hh signaling. Indeed, many mutations that affect cilia formation (e.g. mutation in IFT genes, Kif7) will affect Hh, but this is a consequence of defective cilia, not a cause [8] [9, 10]. Therefore, we focused on the effects of ciliary defects resulting from CCDC66 depletion on Hedgehog pathway activation. We strengthened our conclusions about this process by quantifying the levels of ciliary SMO and its active phosphorylated form, as well as transcriptional activation of Hedgehog target genes under both basal and SAG-activated conditions (Fig. 7A, 7B, 7C).

**Primary cilium and Wnt signaling**:

The impact of canonical Wnt/β-catenin (hereafter Wnt) signaling on primary cilium assembly is complex [11-14]. For example, while WNT3A promotes primary cilium formation in RPE1 and breast cancer cells, hyperactivation of this signaling pathway in other contexts, such as in the mouse neocortex or NIH3T3 cells, inhibits primary cilium formation [15]. Moreover, neither activating nor inhibiting Wnt pharmacologically affected primary cilium formation in several cell lines, including NIH3T3, HEK293, and RPE1 cells [14]. These findings highlight the context-dependent nature and the still unclear mechanisms of Wnt signaling in ciliogenesis.

The relationship between primary cilia and Wnt signaling is not fully understood because directly monitoring a ciliary response to WNT ligands, or experimentally uncoupling ciliary from plasma membrane signaling, has not been feasible [13, 14, 16-19]. Some studies suggest primary cilia may inhibit Wnt signaling, although this is debated. Notably, several key proteins involved in Wnt signaling, such as DVL, FZD, Inversin, and GSK3, localize to the cilium, where they transmit an intraciliary signal that is independent of β-catenin [20].

Given the existing controversies, we have limited our analysis and conclusions to the functions of CCDC66 in Wnt/β-catenin signaling, both within and outside of cilia. Our manuscript provides the first evidence of CCDC66's involvement in Wnt signaling, which may contribute to the epithelial cell organization defects we reported (Fig. 8A, 8B, 8C, 8D). We have added additional data to support the role of CCDC66 in Wnt signaling and more extensively clarified this as response to the Reviewer #3. Moreover, our data suggest that CCDC66's influence on Wnt signaling is β-catenin-dependent, as indicated by observed defects in β-catenin localization and nuclear translocation (Fig. 8E, Fig. 8F). However, it is challenging to determine whether these effects stem from plasma membrane or ciliary signaling, given CCDC66's broad localization within the cell, not limited to cilia. We clarified these conclusions and discussed the existing controversies on the link between primary cilium and Wnt signaling in the Discussion section.

**Minor comments:**
1. Ciliary glycylation has been reported to be involved in regulating cilia length (J Cell Biol. 2017 Sep 4;216(9):2701-2713). Have the authors checked out microtubule glycylation in Ccdc66-depleted cells?

We thank the reviewer for the suggestion. In our revision, we attempted to assess ciliary glycylation levels in control and CCDC66-depleted cells using the anti-Gly-pep1 antibody (AdipoGen AG-25B-0034) [21]. Despite trying multiple fixation methods, our trials with this antibody did not yield robust and specific staining of cilia in IMCD3 cells. Below, we present results from methanol fixation and staining experiments to represent our observations. We also tested the antibody in human RPE-1 cells; however, the results were similar to the IMCD3 cells. This limitation prevented us from assessing the effect of CCDC66 on ciliary glycylation contributing to cilia length phenotypes observed in CCDC66 depletion.

We would like to highlight the original paper that published generation and assessment of glycylation specific antibody [21]. Figures 2C and 2D in that paper show the antibody testing in RPE-1 and IMCD3 cells, and it reports that *“ IMCD3 and RPE-1 cells grew relatively short primary cilia, which were not detected with gly-pep1.”*

2. In Fig. 6E-G, the authors suggest that the localization of the IFT-B complex during ciliary transport is unaffected by Ccdc66 depletion. However, another report (J Cel Biol 2012 Jun 11;197(6):789-800) indicates that the IFT-A complex is required for Shh signaling. Have the authors checked out the localization of the IFT-A complex in Ccdc66-depleted cells?

We thank the reviewer for this suggestion. We have tested several antibodies to evaluate their efficacy in staining ciliary IFT-A components in mouse IMCD3 cells, including IFT-140 (PTG 17460-1-AP), IFT-122 (PTG 19304-1-AP), IFT144 (WDR19; rabbit Proteintech 13647-1-AP) and IFT-121 (WDR35; PTG 26701-1-AP). Only Anti-IFT144 showed satisfactory ciliary positivity (Fig. 7F), while the others failed to stain the cilia in the IMCD3 cells (negative data shown below).

To assess CCDC66’s role on ciliary IFT-A recruitment, we quantified the ciliary levels of IFT-144 in control and CCDC66-depleted cells Our results indicate a significant loss of IFT144 upon CCDC66 depletion, further supporting a decrease in BBSome and defects in Shh signaling (Fig. 7A, 7B, 7C, 7G) [22]. Additionally, defects in retrograde transport could explain the abnormal accumulation of anterograde IFT88 observed in CCDC66-depleted cilia (Fig. 7D). Our analysis of effects of CCDC66 depletion on IFT-A and IFT-B reveals dysfunction in ciliary transport, specifically an imbalance between retrograde and anterograde transport, which might be due to defects in changes in ciliary and cytoplasmic microtubules. This imbalance may lead to the observed defects in cilium length and ciliary signaling.

3. Where is the data in Figure 2D that corresponds to the sentence "Ccdc66-depleted cells... despite apparent ciliary defects..." on page 8?

We thank the reviewer for pointing this out. We have made the correction (please note that the figure labels have changed in the revised manuscript)

4. On page 11, line 8, Fig. 2B should be corrected to Fig. 2C.

We thank the reviewer for pointing this out. We have made the correction (please note that the figure labels have changed in the revised manuscript)

5. Microtubule stability is generally measured by the extent of alpha-tubulin acetylation. Are in vitro assays using alpha-tubulin sufficient to demonstrate microtubule stability? Additionally, in the sample treated with 100nM MBP-mNG Ccdc66, alpha-tubulin appears to be present, but the structure appears to be aggregated. Can this be considered a stable state?

Axonemal stability primarily depends on the intrinsic physical properties of axonemal microtubules and is regulated by tubulin isoforms, post-translational modifications (PTMs), and axoneme-associated proteins such as microtubule-associated proteins (MAPs) [23]. We have recently detailed these factors in a review [24].

To assess whether CCDC66 regulates ciliary stability via acetylation, we measured levels of axonemal acetylation in both control and CCDC66-depleted cells and found no significant differences (Fig. S1I). This rules out a decrease in this post-translational modification as a contributing factor to MT instability.

While acetylation of alpha-tubulin in the microtubule (MT) lumen is a marker of stable microtubules, it is not the only measure of MT stability. Therefore, we used other methods to assay the role of CCDC66 in MT stability. The *in vitro* dilution-induced MT destabilization experiments allowed us to directly assess the impact of CCDC66 on MT stability without the influence of cellular factors. The structures observed in 100 nM MBP-mNG-CCDC66 are not aggregates but bundled MTs, such as the ones in k-fibers [25]. The stability of CCDC66-induced bundles is evidenced by their persistence after the dilution of the microtubule polymerization reaction, whereas control microtubules depolymerized (Fig. 2C).

In addition to *in vitro* assays, we have two additional lines of data that supports the role of CCDC66 in MT stability:

1- We investigated if CCDC66 overexpression enhances ciliary stability by assessing their resistance to microtubule depolymerization in control and CCDC66-expressing cells. High doses of nocodazole lead to shortened, unstable cilia in control cells. However, cells expressing CCDC66 were largely resistant to cilia shortening induced by nocodazole treatment, suggesting that CCDC66 confers greater ciliary stability through microtubule function (Fig. 2A, 2B).
2- In the revised manuscript, we investigated whether microtubule stabilization could reverse the enhanced cilium disassembly observed upon CCDC66 depletion. To this end, we treated both control and CCDC66-depleted ciliated cells with the microtubule-stabilizing drug Taxol upon serum stimulation. We found that this stabilization prevented premature cilia loss in CCDC66-depleted cells (Fig.3D, 3E, 3F), identifying microtubule instability as an underlying mechanism for enhanced disassembly.

6. Images in Fig. S2 show that cilia appear shorter in Flag-miniTurbo-Ccdc66- overexpressed shCtrl cells compared to other figures. For accurate comparison, I suggest showing data from shCtrl cells overexpressing Flag-mini Turbo.

We measured ciliary length separately in wild-type (WT) cells and cells stably expressing miniTurbo-CCDC66, and CEP104-BirA cell lines. In all conditions, we compared the effects of two shRNA conditions: scramble shRNA (control) and Ccdc66 shRNA. The miniTurboID-CCDC66 cells have shorter cilia on average compared to WT cells, whereas the CEP104-BirA cells have longer cilia than WT. The observed effects result from chronic overexpression of these proteins in the cells.

We used miniTurboID-CCDC66 and CEP104-BirA-expressing stable cell lines for phenotypic rescue experiments. Specifically, we assessed their effect on the phenotypes of ciliary shortening and premature ciliary loss during disassembly associated with CCDC66 depletion. Since we transduced each cell line with either shControl or shCCDC66-expressing virus, the resulting phenotypes are comparable and allowed us to conclude that CCDC66 overexpression rescues CCDC66 depletion phenotypes whereas CEP104 cannot (Fig. S2A-F). Therefore, we did not repeat these experiments in miniTurboID-expressing cells, as the effects of CCDC66 depletion on cilium length and disassembly were already established in Figs. 1 and 3.

7. On page 16, Fig. S5D should be corrected to Fig. 5D.

We thank the reviewer for pointing this out. We have made the correction (please note that the figure labels have changed in the revised manuscript)

8. On page 18, it is stated that "the percentage of SMO-positive cilia also decreased", but there appears to be no significant difference between shCtrl and shCcdc66 in the Fig. 6B graph. The data should be confirmed.

We increased the number of experimental replicates from n=2 to n=3. The new data reveal a statistically significant 24% reduction in the percentage of SMO-positive cilia in CCDC66-depleted cells compared to control cells. Additionally, we have incorporated new data into this figure by assessing the levels of activated Smo using a phosphorylated Smo antibody (7TM antibody 7TM0239A, Amerigo Scientific, characterized in [26]. Our results indicate that CCDC66 regulates both the activation of Smo and its targeting to cilia (Fig. 7).

9. In Fig.6C-D, the qRT-PCR data were analyzed using a one-sample t-test, but the error bar range for the shCcdc66 data appear quite wide. Why not analyze the data using other statistics, such as unpaired t-test?

We thank the reviewer for the suggestion. It's true that the qPCR data show considerable variability. In the revised manuscript, we expanded the experiment to include qPCR analysis of target genes under basal conditions and upon SAG activation in both shControl and shCCDC66-treated cells (Fig. 7C). For the analysis, we applied multiple unpaired t-tests with Welch’s corrections, which provided the same significance value as the one-sample t-test (the control DMSO treatment was fixed/normalized to 1 and served as the comparison).

To enhance the statistical confidence of our results throughout the manuscript, we increased the number of experimental replicates from n=2 to n=3 for experiments that initially had only two replicates and applied the appropriate statistical tests.

**Reviwer#2:**
The paper by Deretic et al. on "Ccdc66 regulates primary cilia stability, disassembly and signaling important for epithelial organization" aims to demonstrate the role of the microtubule associated protein (MAP) Ccdc66 in the regulation of cilia disassembly through ectocytosis. It also addresses the role of this protein in Hedgehog and WNT signaling and in epithelial organization. As they stand, the data does not fully support these conclusions. The different observations are also not very well linked together. For all these reasons, I do not think the manuscript is suitable as it stands for publication in Plos Biology.

We thank the reviewer for the feedback. In response to suggestions from all reviewers, we have made substantial revisions to the manuscript and performed many new experiments that strengthen our conclusions, uncover new mechanisms of action, and address any ambiguities. As detailed in our responses to reviewer comments, we believe these extensive revisions clarify our conclusions and more effectively highlight the significance of our findings. Specifically, our findings reveal: 1) the mechanisms regulating cilium stability and length during maintenance and disassembly, and 2) the functions of CCDC66 in ciliary signaling and tissue organization, providing insights into CCDC66-linked ciliopathies.

Regarding the reviewer's comment about the lack of coherence among different observations, we have revised the manuscript text, reorganized the figures, changed the flow of our manuscript, and included a model figure. We believe that these changes clarify our conclusions about both the ciliary and non-ciliary functions and mechanisms of CCDC66.

We summarize below the main conclusions of our manuscript:

1- We show that CCDC66 is required for maintaining the length, stability, and composition of steady-state and disassembling cilia. Fixed and live imaging of cilia during these processes revealed that CCDC66 depletion leads to fluctuations in ciliary length and increased ectocytosis, supporting its role in cilium stability.
2- Phenotypic rescue and *in vitro* experiments demonstrate that CCDC66’s roles in cilium maintenance and disassembly involves stabilization of axonemal microtubules. In cells depleted for CCDC66, enhanced cilium disassembly phenotype was restored by a) inhibiting the AURKA-HDAC6 pathway, which destabilizes axonemal microtubules, b) treatment with taxol, which stabilizes microtubules. Additionally, cilia expressing CCDC66 showed increased resistance to axoneme destabilization induced by microtubule depolymerization. Finally, *in vitro* microtubule stabilization experiments demonstrated that CCDC66 stabilizes microtubules and prevents their dilution-induced disassembly.
3- We identify the first *in vivo* temporal proximity interactome of CCDC66 during cilium disassembly. These maps are the first to chart the interactions during cilium disassembly and provide a valuable resource for the field. By examining previously undescribed proximity interactions in the disassembly proteome, we demonstrated that CCDC66 influences its ciliary functions, either directly or indirectly, by regulating the actin cytoskeleton and RAB-mediated vesicular trafficking at the base of the cilium.
4- We show that CCDC66 is required for regulating cilium composition, ciliary transport by the IFT machinery and BBSome complex, and ciliary signaling through the Hedgehog pathway. We suggest that this reflects the impact of impaired axonemal and cytoplasmic microtubule integrity, which subsequently affects vesicle trafficking and receptor targeting to cilia. Consequently, CCDC66 is necessary for maintaining epithelial cell organization and polarity in 2D and 3D-cultures, and Wnt-signaling pathway, by its ciliary and non-ciliary roles.

To better illustrate our findings, we revised the title of our manuscript to “**CCDC66 cytoskeleton and cilia stability is important for signaling and epithelial organization”**

**Major points:**
1) Depletion of Ccdc66 in serum starved cells resulted in shorter cilia. A phenotype already described in one of the previous manuscript on Ccdc66 by the same laboratory (Odabasi et al.). The authors should address whether the difference in dynamic observed in this steady state cilia or during disassembly is not a consequence of cilia length (Fig.1 and 4). Similarly, the differences observed in the percentage of ciliated cells following serum addition could be a consequence of short cilia disassembling faster than long cilia (Fig.3) and not a specific role of this protein. As it stands it is thus unclear whether Ccdc66 has a function in ciliary disassembly beyond its role in controlling ciliary length.

1- The reviewer makes an excellent point regarding the potential effect of short cilia on faster disassembly. To address this, we compared the cilium disassembly kinetics relative to cilium length by comparing cilia of similar lengths in control and CCDC66-depleted cells at the start of imaging in the GFP-SSTR IMCD3 line (Supplementary Fig. S3, raw data). This analysis revealed that CCDC66-defective cilia fluctuate more in length and shorten more quickly after serum stimulation. Notably, CCDC66-depleted cells exhibited high incidences of instant loss (about %40), while control cilia disassembled mostly through gradual resorption (Fig. 4A, 4B, 4C, S3A). Together, these findings support cilium instability associated with CCDC66 loss.

We include below examples where CCDC66-depleted cilia, similar in length to control cells’ cilia (2 µm), disassemble faster than control upon serum addition. This comparison suggests that CCDC66's influence on cilium disassembly is not solely a result of shorter cilia. Moreover, we calculated the cilium disassembly rates from live imaging videos. CCDC66-depleted cells had a mean disassembly rate of 1.03 ± 0.53 µm/h (n = 30), compared to 0.29 ± 0.13 µm/h (n = 30) in control cells, indicating faster disassembly following CCDC66 depletion.

**Figure Legend**: Cilium disassembly kinetics upon serum stimulation. The IMCD3::SSTR3-GFP cells transduced with either control or Ccdc66 shRNA were grown and serum starved in FluoroDish for 48 h, then imaged with confocal microscopy for 6 h immediately upon serum addition. With images acquired every 4 min. Representative graphs show one control cilium that gradually resorbs, and two CCDC66-depleted cilia that undergo disassembly through instant loss (whole cilium shedding) or gradual resorption.

2- Based on the cellular and *in vitro* data presented in Figures 1-3, we propose that CCDC66 plays a crucial role in ciliary axonemal stability, which contributes to its functions in controlling primary cilium length and disassembly. This is further supported by the results of the following experiment we performed for the revisions (Fig. 3D, 3E, 3F):

We examined whether microtubule stabilization could restore the enhanced cilium disassembly observed following CCDC66 depletion. For this purpose, we treated both control and CCDC66-depleted ciliated cells with the microtubule-stabilizing drug Taxol upon serum stimulation. We then measured the effects of Taxol on ciliation percentage and cilium length to evaluate its impact on CCDC66-related ciliary functions. Our results show that Taxol treatment effectively restored the enhanced disassembly of cilia in CCDC66-depleted cells.

2) It would also be important to block ectocytosis to see whether the change in cilia size are indeed a consequence of the observed increase in this process and to assess the role of Aurora Kinase and HDAC6 in this context.

Below, we detail the specific experiments we conducted and their results.

1- While we interpreted the enhanced ectocytosis in CCDC66-depleted cells as a consequence of cilia instability, the reviewer raises an excellent point about its potential causative role in cilia shortening. To address this, we blocked ciliary ectocytosis and determined whether increased ectocytosis contributes to the changes in cilia size, instability and disassembly (Fig. 6). To block ectocytosis, we used an actin depolymerizer drug based on following studies:

Nager et al. (2017) demonstrated the role of F-actin in ectocytosis by using low doses of the actin depolymerizer drug cytochalasin D to inhibit the process [27]. Similarly, Wang et al. (2019) employed another actin depolymerizer drug, Latrunculin A, to block ectocytosis [28]. Therefore, we examined the phenotypic consequences of inhibiting actin polymerization and, consequently, ciliary ectocytosis, on cilium length and stability during disassembly. Below is summary of our results:

a) CytoD treatment inhibited the enhanced cilium disassembly and shortening mediated by CCDC66 depletion (Fig. 6). These results suggest that CCDC66 modulates cilium disassembly through a process involving actin polymerization.

b) We treated control or CCDC66-depleted IMCD3::GFP-SSTR3 cells with CytoD and monitored their ciliary dynamics using live imaging. CytoD treatment suppressed both cilium disassembly and shortening in these cells (Fig. 6D-E). In cells treated with CytoD, CCDC66-depleted cells exhibited a mean disassembly rate of 0.54±0.35 µm/h (n=30), compared to 1.03 ± 0.53 µm/h (n=30) in untreated cells and 0.35±0.17 µm/h in CytoD-treated control cells (n=30).Interestingly, the fold change in cilium length between control and CCDC66 depletion did not significantly change in CytoD treatment, indicating that actin polymerization is not the only cause of cilia shortening in CCDC66. Importantly, it also minimized the cilium length fluctuations seen with CCDC66 depletion and increased the percentage of intact cilia from about 20% to 50% in CCDC66-depleted cells (Fig. 6F). These results indicate that increased ectocytosis contributes to cilium instability and enhanced disassembly observed in CCDC66-depleted cells.

2- To address the reviewer’s point on assessing the role of Aurora Kinase and HDAC6 for cilium size, we quantified cilium length and percentage in cells treated with MLN or Tubacin and stimulated for cilium disassembly. Treatment with each inhibitor effectively rescued the enhanced cilium disassembly observed in CCDC66-depleted cells 6 h post-serum induction (Fig. 3D-F). Although cilium length was reduced in both control and CCDC66-depleted cells at this time point, neither inhibitor restored the defects in cilium length. These findings indicate that inhibiting the AURKA/HDAC6 pathway activities, such as cilium destabilization through deacetylation, can reverse the disassembly defects associated with CCDC66, but not length defects."

3) Primary-cilium derived extra-cellular vesicles may depend on actin function. Cilia length is also known to depend on actin factors. It will thus be important to address whether Ccdc66 potential interaction with actin actors are involved in its control of ciliary length.

We agree that CCDC66 may indeed regulate actin function in cells and that this might contribute to its ciliary functions. This is supported by the identification of a cluster for actin-binding proteins in the cilium disassembly interactome (Fig. 5D). Moreover, Jijumon et al., 2022 study described CCDC66 as a direct MT-actin cross-linker using in vitro experiments [29]. To investigate the functional relationship between CCDC66 and actin cytoskeleton, we performed the following experiments:

1- We conducted in vitro actin binding experiments, confirming the direct affinity of purified mNG-CCDC66 for actin and demonstrating its effect on altering actin filament organization in vitro (Fig. S5A). Moreover, actin filament abundance and organization were disrupted upon CCDC66 depletion in IMCD3 cells (Fig. S5B)
2- Inhibition of actin polymerization was shown to result in elongated cilia. To determine whether CCDC66 expression impairs CytoD-mediated cilia elongation, we treated IMCD3 cells stably expressing miniTurbo-CCDC66 with CytoD and quantified changes in length of steady-state cilia (Fig. S5C, S5D). We found that CCDC66 expression rescued cilia from over-elongation, further corroborating actin's role in CCDC66-mediated regulation of cilium length.
3- We investigated how inhibition or stabilization of actin polymerization impacts the ciliary phenotypes associated with CCDC66 depletion. To this end, we determined the effect of actin depolymerizer drug, Cytochalasin D, on enhanced cilium disassembly and cilium shortening associated with CCDC66 depletion. The results of these experiments are detailed in our response to this Reviewer’s comment # 2, which together suggest modulation of actin cytoskeleton as a mechanism by which CCDC66 mediates its cellular functions.

To assess the effects of actin stabilization on cilium disassembly, We have tried prolonged (6h) treatment with a low dose of 50 nM jasplakinolide (Jas), an actin stabilizing drug. However, Jas treatment completely detached CCDC66-depleted cells from the substratum and prevented their fixation and imaging.

4- F-actin polymerization at the ciliary tip is a critical step during cilia ectocytosis [30]. Proteins involved in ectocytosis including actin and actin-binding proteins such as RAB7 was shown to localize at ectocytosis sites at the distal end of the cilium [28]. Therefore, we investigated whether CCDC66 localizes at actin-rich ectocytosis sites. We fixed the mNeonGreen-CCDC66 IMCD3 cell line and stained it with phalloidin after 6 hours of serum stimulation. However, we could find only very few instances of F-actin localization at the cilia, which prevented us from drawing conclusions from this experiment. This is likely due to the transient nature of ciliary actin at the ectocytosis sites, which makes it difficult to detect.

Taken together, the experiments mentioned above show that CCDC66 directly binds to actin, and its impact on the actin cytoskeleton is functionally important for its role at the primary cilium. The exact nature of CCDC66 regulation of actin cytoskeleton warrant further detailed cellular and *in vitro* investigation, which would be beyond the scope of this manuscript.

4) Statistics are another major issue in this paper. All experiments have been performed only two times. It is therefore really surprising that the authors can perform a Welch test on the percentage of ciliated cells in each of this two experiments.

We agree with the reviewer's suggestions, and thus added a third experimental replicate for all experiments related to major phenotypes, while also ensuring we used appropriate statistical tests. This includes measurements of ciliary length and percentage, as well as live-cell imaging of ciliary kinetics following CCDC66 depletion. Additionally, we conducted all new experiments targeting actin and ectocytosis, and RABs with three experimental replicates.

5) The result on the Hedgehog pathway have already been published in Odabasi et al on a different cell lines.

We selected IMCD3 cells to examine the role of CCDC66 during Hedgehog pathway activation because they respond more robustly to Sonic hedgehog (Shh) than RPE1 cells. Additionally, our study aims to explore cell-type specific functions of CCDC66. Although not novel, our results further strengthen the role of CCDC66 in this pathway.

While the results on the WNT pathway are confusing according to current literature. It is also unclear whether this is a consequence of Ccdc66 role on ciliary length/ Ectocytosis.

As we discussed in response to Reviewer 1’s point 5, defects in the canonical Wnt/β-catenin pathway may result from ciliary defects associated with CCDC66 depletion. Additionally, our results show disrupted membrane and nuclear localization of β-catenin and defective cell-cell adhesion in CCDC66-depleted cells. These all suggest roles for non-ciliary pools of CCDC66 in the Wnt/β-catenin pathway. Howeover, disentangling the ciliary and non-ciliary functions of CCDC66 within this pathway is challenging, in part due to lack of tools to perturb specific pools of CCDC66 in cells. We have further expanded on this in Discussion section in pgs. 33, 38, 39, 40.

6) Finally, while it is clear that CCDC66 depletion as phenotypic consequences on spheroid and epithelium organization, it is unclear whether it depends on its role in ciliary disassembly, ciliary length or mitotic progression.

We agree with the reviewer on this point. Correlating the diverse functions of CCDC66 with its varied localizations—including centriolar satellites, the centrosome, cilium, and microtubules—is challenging due to the lack of tools to selectively deplete or inactivate it at specific cellular sites. In fact, this is a significant challenge for cellular functional studies. In the Discussion section, we suggested that using non-ciliated Caco-2 and MCF10 cells could help elucidate the distinct contributions of ciliary and mitotic phenotypes to spheroid formation. We hope the reviewer will agree that introducing new cell types is beyond the scope of this paper.

For the revised manuscript, we attempted to assess cell contacts formation in the CEP164-/- IMCD3 cell line, which do not form cilia [31]. However, this was not feasible as the CEP164 knockout also disrupted cell-to-cell contacts, therefore we did not try to form spheroids with these cells. Therefore, distinguishing between the contributions of ciliary and non-ciliary defects to spheroid formation remains challenging for CCDC66. This challenge also applies to other ciliary proteins that have non-ciliary functions. We have included a discussion of these points in the Discussion section in pgs. 39, 40.

Finally, we would like to highlight the additional role of CCDC66 in spheroid formation and the formation/maintenance of cell-to-cell contacts, specifically at adherence junctions (AJs). In CCDC66-depleted cells 2D and 3D cultures, we observed defects in β-catenin membrane and nuclear localization, as well as defects in Wnt pathway activation (Fig. 8). Our new data on deregulation of the endocytic pathway in CCDC66-depleted cells could explain why β-catenin and E-cadherin are not properly targeted and maintained at the cell boundaries (Fig 8E, 8F). While differentiating the contributions of ciliary and non-ciliary functions in spheroid formation upon CCDC66 depletion remains challenging, the role of CCDC66 at cell-contact sites must be considered.

**Reviewer#3:**
In their manuscript, "Ccdc66 regulates primary cilium stability, disassembly and signaling important for epithelial organization," Deretic et al investigate the function of CCDC66, a microtubule associated protein (MAP) in inner medullary collecting duct (IMCD3) kidney epithelial cells. They demonstrate CCDC66 regulates the stability and disassembly of the microtubule axoneme to maintain cilia length through a mechanism involving AurkA and HDAC6. This moves the field forward by revealing a new role for CCDC66 in cilia and places it in a molecular pathway known to regulate the process. Overall, these experiments are thorough, well-controlled, suitably powered and use appropriate statistical analyses so that the data support this conclusion.

We thank the reviewer for recognizing how our manuscript advances the field by revealing how CCDC66 regulates the stability, maintenance, and disassembly of the ciliary axoneme and appreciate the acknowledgement of our thorough, well-controlled experiments and robust statistical analyses that support our conclusions.

I struggle to state the significance of this work however. The problem it purports to solve is "what does CCDC66 do?" But at the end of the manuscript, I am not sure. CCDC66 does not regulate ciliary assembly in IMCD3 cells as the research group previously showed it does in another cell line, retinal pigment epithelial (RPE) cells. While the authors correctly point out the 2 cells lines use distinct ciliary assembly mechanisms (intracellular vs extracellular), it remains unclear whether this explains the difference. There are certainly growing data to show cell type specific roles for cilia-associated proteins. Perhaps CCDC66 has cell type specific roles then. However, the manuscript barely speculates on this point.

1- Regarding the reviewer’s concerns about the uncertainty regarding the cellular functions of CCDC66, this may be due to the way we presented its diverse functions across different experimental approaches within the manuscript’s flow. We summarized the major findings from our study in bullet points in response to Reviewer 2. We revised the flow of our manuscript and included a graphical model (Fig. 9) to illustrate our experimental findings and hypotheses, explained below, more clearly.

Our main findings show that CCDC66 directly stabilizes microtubules (MTs), which are crucial for maintaining ciliary stability. Depletion of CCDC66 results in ciliary instability, leading to shorter and less stable cilia during assembly and maintenance, and enhanced cilia loss during disassembly. Depletion of CCDC66 also results in vesicle trafficking defects that significantly disrupt the accumulation of early and recycling endosomes at the ciliary base. Our observations suggest that these defects in microtubule and vesicle trafficking hinder the proper localization of signaling molecules to the cilia, thereby compromising ciliary function. Furthermore, abnormalities in protein transport interfere with formation of cell-to-cell contacts and disrupt Wnt signaling pathways in CCDC66 depletion. The impairment of signaling through either cilia or cell membranes results in disorganized tissue structure.

2- We appreciate the reviewer highlighting the importance of cell-type-specific ciliary roles for proteins like CCDC66, and we agree that our discussion should have more thoroughly addressed these aspects. In the revised manuscript, we discussed this in more detail (pgs. 4, 7). We note that our research on CCDC66 over the years has investigated its diverse functions across various cell types, with a particular focus on different cellular processes. In this manuscript, we specifically concentrated on the role of CCDC66 in the IMCD3 cell line to explore its potential impact on kidney development, especially regarding primary cilia and tissue organization in both 2D and 3D cultures—areas we were previously unable to investigate using RPE cells.

Instead, the manuscript goes on to use proximity labeling "to identify novel proteins that may regulate ciliary length and stability." Again, the work is well-controlled and thorough. The authors perform the PPL at 2 timepoints during disassembly and compare to an existing dataset in the same cell line when ciliated. However, I am unclear what we learn. There is a list of proteins in proximity to CCDC66 under different conditions (or time points). Some make sense from the literature (so by definition are not novel). None are manipulated to determine whether they are, in fact, novel regulators of ciliary length and/or stability. Furthermore, I am not convinced the authors are correct to focus on the 202 proteins that are shared among the ciliated and disassembling cells. Couldn't CCDC6 only come into proximity of proteins regulating disassembly during disassembly? If so, that would make the 82 proteins shared between the disassembly timepoints and not in the ciliated cells of potential interest to prioritize. Regardless, the PPL represents a great deal of work that is an important starting place for understanding the mechanism of CCDC66 in disassembly. I am sure the authors agree performing such work is outside the scope of this work yet without it, we are left with the manuscript revealing a new role of CCDC66 regulating the stability and disassembly of the microtubule axoneme to maintain cilia length and a long list of proteins that are near CCDC66.

We thank the reviewer for recognizing the well-controlled and thorough nature of our proximity mapping of the CCDC66 interactome during cilium disassembly. We had two primary goals in performing these experiments: 1) To elucidate the mechanisms by which CCDC66 regulates cilium disassembly, and 2) To perform temporal proximity mapping of interactions during cilium disassembly for the first time, providing a valuable resource for the field. Addressing the first objective, we investigated the interaction between CCDC66 and CEP104 during cilium disassembly, as CEP104 was notably enriched during this phase compared to cilium assembly (Fig. S2). However, we did not find a significant relationship. Pursuing the second aim, we analyzed the high-confidence cilium disassembly interactomes and compared them with those from the ciliated state to identify pathways and processes integral to cilium disassembly (Fig. 5, S4). This comparison revealed additional proteins, beyond the expected centrosome, cilium, and satellite proteins, linked to actin, vesicle trafficking, and lipid metabolism as potential regulators of cilium disassembly.

We detailed below how we addressed the major concerns raised by the Reviewer.

1- Regarding the reviewer’s suggestion to focus on unique interactors of CCDC66 during disassembly that are not present during ciliation, we conducted a GO analysis of these 82 proteins. Although we observed enrichment in several categories, such as endocytosis, microtubule cytoskeleton organization and regulation, actin mediated processes and smoothened signaling pathway, the insights gained were limited. We have included this analysis in Table 1/Tab 7 for future reference. However, we derived more substantial insights by focusing on proteins that were upregulated or downregulated during cilium disassembly rather than solely on their presence or absence. By comparing heat maps of steady-state and disassembly interactomes, we decided to concentrate on the actin cytoskeleton and vesicular trafficking.

2- We agree with the reviewer that validating some of these key interactions could significantly enhance our manuscript by revealing new mechanisms by which CCDC66 affects cilium stability, length, and disassembly. To address this, we investigated the role of CCDC66 in regulating ciliary actin and vesicular trafficking, two functional clusters enriched in the CCDC66 disassembly interactome that have not been previously tested in cells. Below, we detail the results from this characterization:

- We chose to focus on actin for two reasons: 1) In vitro experiments have described CCDC66 as a direct microtubule-actin cross-linker [29]. 2) Actin and actin-modulating factors are known to influence ciliogenesis, cilium length, and ciliary ectocytosis. We detailed the experiments we performed for investigating CCDC66-actin relationship in our response to Reviewer#2’s comment 3. Collectively, these experiments defined modulation of actin cytoskeleton as a mechanism by which CCDC66 mediates its functions.

- Two other differentially enriched categories in the CCDC66 cilium disassembly proteome are vesicle trafficking and lipid metabolism, which have been described for their functions during cilium homeostasis and ciliary signaling [32-36]. Moreover, actin and microtubules, cytoskeletal elements regulated by CCDC66, are central to regulation of vesicular trafficking.

Therefore, we investigated whether CCDC66 whether vesicular trafficking near cilia is affected by CCDC66 depletion during both maintenance and disassembly. We first focused on the recycling regulators RAB11 and RAB5, which were enriched in the disassembly proteome and are required for cilia formation and membrane composition (Fig. 5D, S4F, Table 1) [32, 34, 37, 38]. We found a significant reduction of RAB11 and RAB5-positive recycling and early endosomes near the ciliary base in CCDC66-depleted cells, particularly pronounced during cilium disassembly in the case of RAB11 (Fig. S6e, S6F). The total cellular levels of RAB11 and RAB5 did not change upon CCDC66 depletion, confirming that observed changes are not due to their cellular availability (Fig. S6G). These results suggest that CCDC66 regulates early endocytic pathways, thereby promoting the directional trafficking of vesicles to the ciliary base.

Since CCDC66 is involved in ciliary ectocytosis, we also investigated the potential role of CCDC66 in regulating RAB7, the only RAB linked to cilium disassembly so far through inducing ciium ectocytosis [28]. In CCDC66-depleted cells, we observed an increase in RAB7-positive vesicles near the ciliary base, contrasting with reductions in RAB11 and RAB5 (Fig. 6SA-D). This suggests that disruption of apical endocytosis could lead to an increase in RAB7-positive vesicles marking activated protein degradation pathway. Notably, we detected a few instances of RAB7 accumulation within CCDC66-depleted cilia, leading to bending and decapitation (Fig S6H). This might explain the increased ectocytosis observed with CCDC66 depletion.

Collectively, our results highlight CCDC66's role in regulating the actin cytoskeleton and various stages of vesicular trafficking, suggesting potential new mechanisms for its cellular functions. We highlight that this is the first report linking CCDC66 with vesicular trafficking, though we do not aim to provide a complete mechanistic insight into this regulation and its functional relevance. Future studies will be crucial for further exploring this new function of CCDC66, which will likely have significant implications for its ciliary and non-ciliary roles.

Finally, the manuscript examines some functional consequences of CCDC66 depletion. It argues that CCDC66 depletion results in dampened Hh and Wnt response. In the Hh work, the authors need to show the Gli1 and Ptch1 levels in unstimulated as well as stimulated conditions. They use SAG which directly binds and activates SMO to stimulate yet also show a defect in SMO ciliary enrichment. This is confusing. First, I acknowledge that ciliary SMO enrichment does not correlate to pathway activation. But if CCDC66 is regulating that enrichment, it appears that CCDC66 acts upstream of SMO activation. If so, then SAG stimulation should overcome that function. Are the data arguing that CCDC66 regulated Hh at a step upstream of SMO and an additional step downstream of activated SMO? The authors could try the new phosphorylated SMO antibody characterized in PMID: 37214942 as it is commercially available and could at least get at whether CCDC66 regulates the activation of SMO.

We thank the reviewer for the excellent suggestions on further exploring the role of CCDC66 in Hedgehog signaling. We agree with the reviewer’s concerns regarding the lack of data on how CCDC66 depletion affects Hedgehog transcriptional activation in unstimulated cells and how it regulates the Hedgehog pathway. To address these, we performed additional experiments which together strengthened the role of CCDC66 and its microtubule-stabilizing activity in Hedgehog pathway activation. Below are the details of these experiments:

1- We added qPCR data on Gli1 and Ptch1 levels in unstimulated cells and show that mRNA levels are decreased in unstimulated and stimulated CCDC66-depleted cells (Fig. 7C).

2- We have also investigated activation of SMO in IMCD3 cells using anti-phospho Smo antibody (7TM antibody 7TM0239A, Amerigo Scientific, characterized in [26]). Our new results suggest that CCDC66 functions upstream of SMO activation, as the ciliary levels of phospho-SMO are significantly reduced, similar to the decrease observed in SMO itself (Fig 7A, 7B). We think that even SAG treatment and activation of SMO at the plasma membrane cannot overcome this, as vesicle trafficking is the main mechanism of SMO delivery to the cilia and it is significantly affected in CCDC66-depleted cells (Fig. S6). In addition, ciliary stability and structure are crucial for proper ciliary Smo levels and downstream activity. Downstream effects similar to those observed have also been reported with mutations in IFT172, IFT88, KIF3A, and KIF7 [7, 8, 10]. Given this background, it was logical for us to assess SMO ciliary levels, followed by testing SMO activity through downstream transcriptional targets of the Hh pathway, including levels of Gli and Ptch mRNA, which are part of a feedback loop.

3- We measured Smo ciliary accumulation after 4 hours of SAG treatment in cilia depleted of CCDC66 following Taxol treatment to stabilize microtubules (Fig. S6K Strikingly, taxol treatment partially restored the defects in ciliary recruitment of SMO, suggesting that defects in stability of microtubules partially contribute to the Hedgehog pathway defects associated with CCDC66 loss.

In the Wnt work, the authors need to acknowledge the everchanging and controversial relationship of cilia and Wnt signaling. And they need to provide a much clearer, data-based framework from which to interpret these experiments. Are IMCD3 cells Wnt responsive? What Wnt ligand? What are the transcriptional targets (Axin2 seems highly likely but are the others know in IMCD3 cells)? And again, the authors need to show the Axin2, Fibronectin and Pdk1 transcript levels in unstimulated as well as stimulated conditions.

The IMCD3 cells are Wnt responsive and the effects of Wnt signaling were previously tested in IMCD3 [14]. For initial submission, we did not use Wnt ligand but measured basal levels of Wnt activity in control and CCDC66-depleted cells. In the revised manuscript, we examined Wnt transcriptional response with and without treatment of a Wnt agonist. Specifically, we used the Wnt Agonist, BML-284, that increases pathway activity 4-5-fold in higher doses (Lie et al., 2005 PMID: 15724259). We performed quantitative PCR analysis on Axin 2, Fibronectin, and PDK1 with and without agonist treatment. In these experiments, BML-284 stimulation upregulated the expression of Axin2, Pdk1, and Fibronectin in control cells. However, CCDC66-depleted cells showed defects in the expression of Axin2, Pdk1, and Fibronectin compared to control cells under both basal and BML-284 stimulated conditions (Fig. 8G). These results indicate a disruption in Wnt pathway activation due to CCDC66 depletion."

Regarding the reviewer's question about our choice of targets, as noted, Axin 2 is the most responsive downstream target of β-catenin. We selected fibronectin due to its importance in epithelial cell attachment and tissue organization, which is explored in Fig. (8A, 8B, 8C, 8D). Given the observed cell death in CCDC66-depleted cells (Fig. 8D) and its potential relation to WNT signaling, we also tested the metabolic target of the pathway, PDK1.

Finally, as the reviewer noted, the role of cilia in Wnt signaling and the influence of Wnt signaling on cilia formation is more controversial. We provided a detailed explanation in response to Reviewer #1’s Comment 5. Additionally, we highlighted this controversy, with a focus on how CCDC66 might regulate this pathway through its ciliary and non-ciliary pools, with the following text in the Discussion section in pg. 33, 39, 40.

**Minor points:**
Supp Fig 1 shows specificity of Ab, and also shows heterogeneity in KD by siRNA, which is to be expected for the technique. This is clear in MB staining (which I assume is midbody) where CCDC66 staining persistence is visible in top right of green inset. Additionally, there is green signal overlapping the acTub staining on the left side of the shCcdc66 panel that looks ciliary. Broader point is that it appears that the depletion overall is strong but varies cell by cell making it likely that much of the variability in phenotype may be driven by the KD technique. This should be mentioned. At one level, that further supports the conclusion that CCDC66 regulates the stability and disassembly of the microtubule axoneme - but in terms of signaling and broader functional consequences, the variability make the data challenging to interpret.

We agree with the reviewer that we could not completely deplete CCDC66 in every cell, which is a common limitation of knockdown techniques such as shRNA or siRNA. Indeed, our intention was to illustrate this heterogeneity with Fig. 1 and Supplementary Fig. 1, and we appreciate the reviewer’s recognition of this. We included a mention of this point on page X of the Results section as follows:

*“The loss of CCDC66 ciliary and previously reported midbody signals in cells transduced with Ccdc66 shRNA compared to control cells validates the specificity of the rat CCDC66 antibody (Fig. S1A) [25]. However, there was variation in its depletion efficiency across different cells (Fig. S1A).”*

In loss-of-function experiments, where antibody combinations were possible, we used our custom-made anti-CCDC66 antibody, raised in rats, to identify CCDC66-depleted cells. To this end, we applied a threshold for the CCDC66 signal; measurements from cells above this threshold were excluded from our analysis. Specifically, we applied this thresholding approach to quantify the ciliary levels of proteins involved in transport (IFT88, IFT144, BBS4) and signaling (SMO), addressing the reviewer's concerns about variability. We added this experimental detail to the Methods section under 'Microscopy and Image Analysis.

In methods, description of In vitro microtubule stabilization assay is confusing. It sounds like there was no GTP added to buffers with the indicated proteins (100 nM of protein His-MBP-mNeonGreen, His-MBP- mNeonGreen-Ccdc66 or His-MBP-TOGARAM1TOG1TOG2). The text of the manuscript suggests there is GTP. This needs to be clarified for the experiment to be interpretable.

The absence of GTP led to the destabilization of the diluted microtubules, resulting in their depolymerization. To clarify this point, we revised the sentence in the Results section as follows:

“*Polymerized rhodamine-labeled microtubules were diluted 10-fold in prewarmed buffer lacking GTP and glycerol, with and without the indicated proteins, and the reaction was imaged in a flow chamber.*”

The authors should specify the full timeline of the various manipulations. They state that the shRNA treatment requires 6 days. They also serum starve and serum starve then serum stimulate. Is this all sequential? In other words, are there instances when the serum starvation starts prior to the 6 days of shRNA treatment? Or other overlap. It needs to be clear for others to repeat.

We thank the pointing the lack of clarity and thus added methodological details to both the results section, including experimental diagrams in all figures 1E, 2A, 3C, 4A, 4D, 6B, 6D, 7A, 8G, S4A, S6K and the methods section.

To address the reviewer’s questions, in our methodology, we always performed shRNA treatment prior to serum starvation. 6 days of shRNA treatment was necessary for efficient depletion of CCDC66, which was verified by immunoblotting and immunofluorescence (Fig. S1A, S1B). We infected cells with viruses carrying scramble or CCDC66 shRNA, followed by puromycin selection. After the cells recovered from the antibiotic selection, we started experiments involving serum starvation to observe cilia or serum add-back after 48h serum starvation to observe cilia disassembly. Although this would lead to the generation of stable cell lines every time, we have not used these for a prolonged period (high cell passage number) due to the negative effect of CCDC66 deletion on mitosis and overall cell viability. Prolonged time in culture was positively selecting cells less depleted or not depleted of CCDC66.

In the abstract, I believe the authors broad point, that cilia disassembly is less well understood than cilia assembly is true. However, many would take issue with the statement that "cilium assembly is well-understood", most notably those who study it and possess many questions. They deserve a modest rephrasing.

We agree with the reviewer and rephased the sentence in the Abstract as follows:

“*The mechanisms by which a cilium maintains its stability and length during its life cycle are not well understood.*”

Overall, the manuscript brings in a great deal of the literature yet there are quite a few instances where references are missing. This will simply require a rigorous proofread.

We thank the reviewer for this comment. We thoroughly reviewed and hopefully appropriately acknowledged the relevant literature in the revised manuscript, with 100 references.

On page 17 of the manuscript, the authors state, "Although inhibiting AurkA and HDAC6 rescued Ccdc66 disassembly phenotypes, our analysis did not detect these key players in cilium disassembly interactome. This indicates that Ccdc66 has an indirect relationship with these proteins." The final statement is inaccurate as PPL not picking up a protein only means that it did not pick up that protein. There are many reasons direct interactors may not be detected at specific times or under specific conditions using PPL-based methods.

We agree with the reviewer on this point. Since the revised manuscript emphasizes the characterization of new relationships revealed by the proximity interactome, rather than a detailed interpretation, we removed that section. Instead, we concentrated on explaining how we validated the interactome and why we chose actin and vesicular trafficking for further exploration (pgs. 24-27).

It would be far easier to follow when the authors are discussing protein or transcript if the authors adhered to nomenclature guidelines of mammalian proteins being in capital letters and genes being in italics (and caps in the case of human).

We thank the reviewer for pointing out the correct nomenclature guidelines for mammalian proteins. We revised protein names to all uppercase letters, leaving RNA nomenclature in lowercase.

**References**

1.	Mirvis M, Stearns T, James Nelson W. Cilium structure, assembly, and disassembly regulated by the cytoskeleton. Biochem J. 2018;475(14):2329-53. doi: 10.1042/BCJ20170453. PubMed PMID: 30064990; PubMed Central PMCID: PMCPMC6068341.

2.	Pedersen LB, Schroder JM, Satir P, Christensen ST. The ciliary cytoskeleton. Compr Physiol. 2012;2(1):779-803. doi: 10.1002/cphy.c110043. PubMed PMID: 23728985.

3.	Sorokin S. Centrioles and the formation of rudimentary cilia by fibroblasts and smooth muscle cells. J Cell Biol. 1962;15:363-77. PubMed PMID: 13978319; PubMed Central PMCID: PMCPMC2106144.

4.	Sorokin SP. Reconstructions of centriole formation and ciliogenesis in mammalian lungs. J Cell Sci. 1968;3(2):207-30. Epub 1968/06/01. PubMed PMID: 5661997.

5.	Molla-Herman A, Ghossoub R, Blisnick T, Meunier A, Serres C, Silbermann F, et al. The ciliary pocket: an endocytic membrane domain at the base of primary and motile cilia. J Cell Sci. 2010;123(Pt 10):1785-95. doi: 10.1242/jcs.059519. PubMed PMID: 20427320.

6.	Ganga AK, Kennedy MC, Oguchi ME, Gray S, Oliver KE, Knight TA, et al. Rab34 GTPase mediates ciliary membrane formation in the intracellular ciliogenesis pathway. Curr Biol. 2021;31(13):2895-905 e7. Epub 2021/05/15. doi: 10.1016/j.cub.2021.04.075. PubMed PMID: 33989527; PubMed Central PMCID: PMCPMC8282722.

7.	Bangs F, Anderson KV. Primary Cilia and Mammalian Hedgehog Signaling. Cold Spring Harb Perspect Biol. 2017;9(5). doi: 10.1101/cshperspect.a028175. PubMed PMID: 27881449; PubMed Central PMCID: PMCPMC5411695.

8.	He M, Subramanian R, Bangs F, Omelchenko T, Liem KF, Jr., Kapoor TM, et al. The kinesin-4 protein Kif7 regulates mammalian Hedgehog signalling by organizing the cilium tip compartment. Nat Cell Biol. 2014;16(7):663-72. Epub 2014/06/24. doi: 10.1038/ncb2988. PubMed PMID: 24952464; PubMed Central PMCID: PMCPMC4085576.

9.	Cheung HO, Zhang X, Ribeiro A, Mo R, Makino S, Puviindran V, et al. The kinesin protein Kif7 is a critical regulator of Gli transcription factors in mammalian hedgehog signaling. Sci Signal. 2009;2(76):ra29. Epub 2009/06/25. doi: 10.1126/scisignal.2000405. PubMed PMID: 19549984.

10.	Endoh-Yamagami S, Evangelista M, Wilson D, Wen X, Theunissen JW, Phamluong K, et al. The mammalian Cos2 homolog Kif7 plays an essential role in modulating Hh signal transduction during development. Curr Biol. 2009;19(15):1320-6. Epub 2009/07/14. doi: 10.1016/j.cub.2009.06.046. PubMed PMID: 19592253.

11.	Berbari NF, O'Connor AK, Haycraft CJ, Yoder BK. The primary cilium as a complex signaling center. Curr Biol. 2009;19(13):R526-35. Epub 2009/07/16. doi: 10.1016/j.cub.2009.05.025

S0960-9822(09)01126-9 [pii]. PubMed PMID: 19602418.

12.	Lee KH. Involvement of Wnt signaling in primary cilia assembly and disassembly. FEBS J. 2020;287(23):5027-38. Epub 20201019. doi: 10.1111/febs.15579. PubMed PMID: 33015954.

13.	Niehrs C, Da Silva F, Seidl C. Cilia as Wnt signaling organelles. Trends Cell Biol. 2024. Epub 2024/05/03. doi: 10.1016/j.tcb.2024.04.001. PubMed PMID: 38697898.

14.	Zhang K, Da Silva F, Seidl C, Wilsch-Brauninger M, Herbst J, Huttner WB, et al. Primary cilia are WNT-transducing organelles whose biogenesis is controlled by a WNT-PP1 axis. Dev Cell. 2023;58(2):139-54 e8. Epub 2023/01/25. doi: 10.1016/j.devcel.2022.12.006. PubMed PMID: 36693320.

15.	Song L, Jia Y, Zhu W, Newton IP, Li Z, Li W. N-terminal truncation mutations of adenomatous polyposis coli are associated with primary cilia defects. Int J Biochem Cell Biol. 2014;55:79-86. Epub 2014/08/26. doi: 10.1016/j.biocel.2014.08.010. PubMed PMID: 25150829.

16.	Corbit KC, Shyer AE, Dowdle WE, Gaulden J, Singla V, Chen MH, et al. Kif3a constrains beta-catenin-dependent Wnt signalling through dual ciliary and non-ciliary mechanisms. Nat Cell Biol. 2008;10(1):70-6. Epub 20071216. doi: 10.1038/ncb1670. PubMed PMID: 18084282.

17.	Lancaster MA, Schroth J, Gleeson JG. Subcellular spatial regulation of canonical Wnt signalling at the primary cilium. Nat Cell Biol. 2011;13(6):700-7. Epub 2011/05/24. doi: 10.1038/ncb2259. PubMed PMID: 21602792; PubMed Central PMCID: PMCPMC3107376.

18.	Stypulkowski E, Feng Q, Joseph I, Farrell V, Flores J, Yu S, et al. Rab8 attenuates Wnt signaling and is required for mesenchymal differentiation into adipocytes. J Biol Chem. 2021;296:100488. Epub 20210301. doi: 10.1016/j.jbc.2021.100488. PubMed PMID: 33662399; PubMed Central PMCID: PMCPMC8042397.

19.	Veland IR, Montjean R, Eley L, Pedersen LB, Schwab A, Goodship J, et al. Inversin/Nephrocystin-2 is required for fibroblast polarity and directional cell migration. PLoS One. 2013;8(4):e60193. Epub 20130408. doi: 10.1371/journal.pone.0060193. PubMed PMID: 23593172; PubMed Central PMCID: PMCPMC3620528.

20.	Niehrs C, Da Silva F, Seidl C. Cilia as Wnt signaling organelles. Trends Cell Biol. 2025;35(1):24-32. Epub 20240501. doi: 10.1016/j.tcb.2024.04.001. PubMed PMID: 38697898.

21.	Gadadhar S, Dadi H, Bodakuntla S, Schnitzler A, Bieche I, Rusconi F, et al. Tubulin glycylation controls primary cilia length. J Cell Biol. 2017;216(9):2701-13. Epub 2017/07/09. doi: 10.1083/jcb.201612050. PubMed PMID: 28687664; PubMed Central PMCID: PMCPMC5584158.

22.	Liem KF, Jr., Ashe A, He M, Satir P, Moran J, Beier D, et al. The IFT-A complex regulates Shh signaling through cilia structure and membrane protein trafficking. J Cell Biol. 2012;197(6):789-800. Epub 2012/06/13. doi: 10.1083/jcb.201110049. PubMed PMID: 22689656; PubMed Central PMCID: PMCPMC3373400.

23.	Orbach R, Howard J. The dynamic and structural properties of axonemal tubulins support the high length stability of cilia. Nat Commun. 2019;10(1):1838. Epub 2019/04/25. doi: 10.1038/s41467-019-09779-6. PubMed PMID: 31015426; PubMed Central PMCID: PMCPMC6479064.

24.	Deretic J, Odabasi E, Firat-Karalar EN. The multifaceted roles of microtubule-associated proteins in the primary cilium and ciliopathies. Journal of Cell Science. 2023;136(23). doi: ARTN jcs261148

10.1242/jcs.261148. PubMed PMID: WOS:001163814400002.

25.	Batman U, Deretic J, Firat-Karalar EN. The ciliopathy protein CCDC66 controls mitotic progression and cytokinesis by promoting microtubule nucleation and organization. PLoS Biol. 2022;20(7):e3001708. Epub 2022/07/19. doi: 10.1371/journal.pbio.3001708. PubMed PMID: 35849559; PubMed Central PMCID: PMCPMC9333452.

26.	Walker MF, Zhang J, Steiner W, Ku PI, Zhu JF, Michaelson Z, et al. GRK2 Kinases in the Primary Cilium Initiate SMOOTHENED-PKA Signaling in the Hedgehog Cascade. bioRxiv. 2024. Epub 2023/05/22. doi: 10.1101/2023.05.10.540226. PubMed PMID: 37214942; PubMed Central PMCID: PMCPMC10197709.

27.	Nager AR, Goldstein JS, Herranz-Perez V, Portran D, Ye F, Garcia-Verdugo JM, et al. An Actin Network Dispatches Ciliary GPCRs into Extracellular Vesicles to Modulate Signaling. Cell. 2017;168(1-2):252-63 e14. doi: 10.1016/j.cell.2016.11.036. PubMed PMID: 28017328; PubMed Central PMCID: PMCPMC5235987.

28.	Wang G, Hu HB, Chang Y, Huang Y, Song ZQ, Zhou SB, et al. Rab7 regulates primary cilia disassembly through cilia excision. Journal of Cell Biology. 2019;218(12):4030-41. doi: 10.1083/jcb.201811136. PubMed PMID: WOS:000500574100018.

29.	Jijumon AS, Bodakuntla S, Genova M, Bangera M, Sackett V, Besse L, et al. Lysate-based pipeline to characterize microtubule-associated proteins uncovers unique microtubule behaviours. Nat Cell Biol. 2022;24(2):253-67. Epub 2022/02/02. doi: 10.1038/s41556-021-00825-4. PubMed PMID: 35102268.

30.	Phua SC, Chiba S, Suzuki M, Su E, Roberson EC, Pusapati GV, et al. Dynamic Remodeling of Membrane Composition Drives Cell Cycle through Primary Cilia Excision. Cell. 2017;168(1-2):264-79 e15. Epub 2017/01/14. doi: 10.1016/j.cell.2016.12.032. PubMed PMID: 28086093; PubMed Central PMCID: PMCPMC5660509.

31.	Mick DU, Rodrigues RB, Leib RD, Adams CM, Chien AS, Gygi SP, et al. Proteomics of Primary Cilia by Proximity Labeling. Dev Cell. 2015;35(4):497-512. doi: 10.1016/j.devcel.2015.10.015. PubMed PMID: 26585297; PubMed Central PMCID: PMC4662609.

32.	Blacque OE, Scheidel N, Kuhns S. Rab GTPases in cilium formation and function. Small GTPases. 2018;9(1-2):76-94. Epub 2017/10/27. doi: 10.1080/21541248.2017.1353847. PubMed PMID: 29072526; PubMed Central PMCID: PMCPMC5902210.

33.	Jewett CE, Soh AWJ, Lin CH, Lu Q, Lencer E, Westlake CJ, et al. RAB19 Directs Cortical Remodeling and Membrane Growth for Primary Ciliogenesis. Dev Cell. 2021;56(3):325-40 e8. Epub 2021/02/10. doi: 10.1016/j.devcel.2020.12.003. PubMed PMID: 33561422; PubMed Central PMCID: PMCPMC7880532.

34.	Kim J, Lee JE, Heynen-Genel S, Suyama E, Ono K, Lee K, et al. Functional genomic screen for modulators of ciliogenesis and cilium length. Nature. 2010;464(7291):1048-51. Epub 2010/04/16. doi: 10.1038/nature08895. PubMed PMID: 20393563; PubMed Central PMCID: PMCPMC2929961.

35.	Stuck MW, Chong WM, Liao JC, Pazour GJ. Rab34 is necessary for early stages of intracellular ciliogenesis. Curr Biol. 2021;31(13):2887-94 e4. Epub 20210513. doi: 10.1016/j.cub.2021.04.018. PubMed PMID: 33989524; PubMed Central PMCID: PMCPMC8282653.

36.	Westlake CJ, Baye LM, Nachury MV, Wright KJ, Ervin KE, Phu L, et al. Primary cilia membrane assembly is initiated by Rab11 and transport protein particle II (TRAPPII) complex-dependent trafficking of Rabin8 to the centrosome. Proc Natl Acad Sci U S A. 2011;108(7):2759-64. Epub 2011/01/29. doi: 10.1073/pnas.1018823108. PubMed PMID: 21273506; PubMed Central PMCID: PMCPMC3041065.

37.	Knodler A, Feng S, Zhang J, Zhang X, Das A, Peranen J, et al. Coordination of Rab8 and Rab11 in primary ciliogenesis. Proc Natl Acad Sci U S A. 2010;107(14):6346-51. Epub 20100322. doi: 10.1073/pnas.1002401107. PubMed PMID: 20308558; PubMed Central PMCID: PMCPMC2851980.

38.	Zhai D, Li L, Wang D, Wang W, Zhao S, Wang X, et al. TBC1D20 coordinates vesicle transport and actin remodeling to regulate ciliogenesis. J Cell Biol. 2025;224(4). Epub 20250127. doi: 10.1083/jcb.202406139. PubMed PMID: 39868814; PubMed Central PMCID: PMCPMC11781271.
