# Supplementary material for: CCDC66 regulation of cytoskeleton and cilia stability is important for signaling and epithelial organization
Source: PLoS Biol. 2025 Jul 29;23(7):e3003313. doi: 10.1371/journal.pbio.3003313 (PMC12324684; doi:10.1371/journal.pbio.3003313)
Supplement: Attachment — Submitted filename: PLOS Biology revision comments 2.docx [file pbio.3003313.s012.docx]

I have thoroughly reviewed the authors' rebuttal letter, revised manuscript text, figures, and supplementary materials. The authors have comprehensively and convincingly addressed my comments, Specifically:

1. The authors provided experimental evidence differentiating roles of CCDC66 in ciliogenesis, axoneme stabilization, and disassembly processes, addressing my initial concerns.
2. Additional data regarding ectocytosis and the involvement of the actin cytoskeleton and microtubule stabilization provided substantial mechanistic clarity.
3. The statistical robustness of the data has improved, fulfilling all requested criteria.
4. Revised explanations and additional data on the Hedgehog and Wnt signaling pathways further clarified the manuscript's conclusions.
5. The graphical model and explicit discussion added by the authors now effectively convey the integration of their findings and potential physiological relevance.

Overall, the revisions have substantially strengthened the manuscript, resolving all the concerns raised in the previous round of review.

Therefore, I am pleased to recommend acceptance of this manuscript for publication in PLOS Biology.
